# Looking Competent Does Not Appeal to All Voters Equally: The Role of Social Class and Politicians’ Facial Appearance for Voting Likelihood

**DOI:** 10.1177/01461672231181465

**Published:** 2023-07-07

**Authors:** Fabienne Unkelbach, Tatjana Brütting, Nina Schilling, Michaela Wänke

**Affiliations:** 1 University of Mannheim, Mannheim, Germany; 2 Zeppelin University, Friedrichshafen, Germany

**Keywords:** political psychology, appearance-based politics, big two, voting behavior, self-concept

## Abstract

Voters generally value competence in politicians. Four studies, all conducted in Germany, show that this is especially pronounced in people of higher compared with lower social class. The first study, with a representative sample (*N*_1_ = 2239), found that the reported importance of competence in politicians increased with increasing socioeconomic status (SES). This was mediated by self-perceived competence which was higher in participants of higher SES. In three further studies (two preregistered, *N*_2a&2b_ = 396, *N*_3_ = 400) participants merely saw pictures of politicians’ faces. Perceived competence based on facial appearance increased the likelihood of voting for a politician. Again, this effect was stronger among participants of higher compared with lower SES. This moderation persisted after controlling for participants’ political orientation and politicians’ perceived warmth and dominance. We discuss implications for future research on the psychological underpinnings of social class as well as appearance effects in the political context.

Voting decisions are to some extent influenced by candidates’ presumed personality characteristics (Funk, 1999; Kinder et al., 1980; Laustsen & Bor, 2017; Miller et al., 1986; Olivola & Todorov, 2010a; Todorov et al., 2005). Especially perceived competence appears to be relevant for voters. Voters’ ratings of the perceived competence of political candidates were positively correlated with their overall evaluation (Funk, 1999; Miller et al., 1986) and with voting for them (Laustsen & Bor, 2017). Even in the absence of further information merely looking competent seems important for electoral success (Antonakis & Dalgas, 2009; Ballew & Todorov, 2007; Olivola & Todorov, 2010a, 2010b; Todorov et al., 2005, Todorov et al., 2015). For example, in Todorov et al.’s (2005) seminal study, participants’ competence judgments based on portraits of candidates predicted not only the actual election outcome but also the difference in votes between candidates.

Despite numerous evidence for the importance of perceived competence, one may wonder whether competence is equally relevant for all voters. We argue that the importance of politicians’ perceived competence for voting likelihood depends on voters’ social class with those of higher social class valuing competence more.

## Why Competence May Appeal More to Members of High Social Class

Before we delineate our hypothesis a definition of the key concepts is in order. Social class can be conceptualized by objective components that describe the material resources an individual possesses (Kraus et al., 2012) such as financial resources, educational attainment and occupational prestige. We refer to these as objective socioeconomic status (SES). Previous research shows that SES is linked with individuals’ perceived rank in society in comparison to others, a concept often referred to as subjective social status (SSS; Adler et al., 2000; Kraus et al., 2013). While SSS and SES tend to be moderately positively correlated, SSS goes beyond SES by capturing social comparison processes aside from objective assessments (e.g., Tan et al., 2020).

With regard to our second central concept, competence, we follow the concepualization of the Big Two framework (Abele et al., 2016; Abele & Wojciszke, 2007, 2014) which postulates two main content dimensions of social judgment, namely agency and communion. In this model, competence is conceptualized as one subfacet of agency and defined as the ability to accomplish tasks, i.e., intelligence and skill. In contrast, assertiveness, the second facet of agency, is understood as one’s motive to promote the self, i.e., ambitiousness and self-confidence. Communion, on the other hand, encompasses the two subfactes warmth (reflecting empathy and likeability) and trustworthiness (reflecting sincerity and honesty).

Importantly, stereotypes of high and low status groups differ on these dimensions. High-status people are believed to be more competent than low-status people (Cuddy et al., 2008; Fiske et al., 2002, 2007). This stereotype of higher competence of high status groups seems to be shared in many societies (Durante et al., 2013) and, crucial for our argument, is shared by high status people about themselves (Abele, 2003; Belmi et al., 2020; Oldmeadow & Fiske, 2010). Assuming that competence plays a larger role in the self-concept of high- compared with low-status people (see Kraus et al., 2012) we argue that this trait can be considered as chronic self-relevant knowledge, i.e., a schematic trait.

Schematic traits guide the processing of information about oneself and about others (Fong & Markus, 1982; Markus, 1977; Shoda & McConnell, 2013). When perceiving others people primarily attend to information related with their own self-schema (Fong & Markus, 1982; Riggs & Cantor, 1984) and weigh these information more heavily when forming an impression of the target (Carpenter, 1988; Sedikides & Skowronski, 1993). Indeed, people from a higher social class distinguish others especially with regard to their perceived competence (Oldmeadow & Fiske, 2010). Accordingly, one may expect that higher-class voters base their evaluation of politicians more on perceived competence than lower-class voters.

This assumption is in line with findings that voters generally evaluate politicians more positively when they share personality characteristics (Caprara et al., 2007; Caprara & Zimbardo, 2004). As higher-class voters feel more competent, they may perceive candidates who appear more competent as more similar.

Specifically, we argue that people of higher SES are competence-schematics and should therefore value competence in politicians more than people of lower SES. People of higher SES share specific experiences during their socialization in an environment that is determined by educational and financial achievement (e.g., Stephens et al., 2014), and as competence is instrumental to both the concept is omnipresent and easily accessible. These experiences might foster a class-specific tendency to establish competence as schematic trait and distinguish and evaluate others accordingly.

For SSS, we do not have a firm hypothesis regarding a preference for competence. One may assume that people who think of themselves as high in social status also ascribe themselves the attributes stereotypically ascribed to high status people. This assumption would predict similar effects for SES and SSS if self-schema is responsible for the effects.

However, it is unclear whether the self-schema of people high in SSS parallels that of people high in SES. It is noteworthy that previous studies on the importance of candidate traits found diverging effects of SES and (manipulated or chronic) SSS (Callaghan et al., 2022; Tan & Kraus, 2018).

For our main hypothesis that voters high in SES weigh competence in a politician more heavily than lower-class voters there is already some supporting evidence. Analyses of data from the American National Election Studies (ANES) show that voters with higher educational attainment were more likely to mention competence when asked to name aspects that might make them vote for/against presidential candidates than less educated voters (Miller et al., 1986). Importantly, this group of voters was more likely to state that competence (vs. warmth) is an important characteristic in an ideal U.S. president (Kinder et al., 1980). Likewise, a more recent analysis of ANES data (Laustsen & Bor, 2017; Table SI.11b) reveals a significant interaction between voters’ educational level and politicians’ perceived competence on vote choice in the proposed direction.^1^

Moreover, Callaghan et al. (2022) presented descriptions of politicians in form of word clouds expressing competence or warmth. Compared with participants of lower SES those of higher SES indicated a higher voting likelihood for the candidate presented as competent and were more likely to prefer the competent over the warm candidate. The present research goes beyond the previous evidence by not only relying on self-reported importance but moreover by testing whether the effect manifests for the impact of competence perceived from facial appearance on voting preferences.

## The Present Research

Four studies tested the hypothesis that the perceived competence of a political candidate has a larger impact on the propensity of voting for this candidate among voters with higher SES.

In contrast to previous research which has focused on U.S. samples, we investigate the association between social class and important candidate traits in the German context. Beyond reasons of external validation, a conceptual replication outside the United States is particularly relevant in this case as the societal structure of the United States may have contributed to the previously observed effects.

The fact that people of higher social class consider competence as more important than people of lower social class may be particularly pronounced in the United States for two reasons: First, competence is a trait which is specifically valued in individualistic cultures. Within a culture people who excel on the dimensions valued in this culture are more likely to ascend to higher positions and in turn may hold those values particularly dear (Gobel & Miyamoto, 2022). Thus, the association of higher status with more individualistic traits is particularly pronounced in individualistic cultures (Gobel & Miyamoto, 2022; Zhang et al., 2021). It should be noted that although Germany is more individualistic than collectivistic (Hofstede et al., 2010), it is much less individualistic than the United States, the most individualistic country in the world. Possibly the association between social class and a preference for competence is stronger in the United States due to its extreme position on the individualism-collectivism scale. In Germany, as a less individualistic society, social classes may differ less in how they value such individualistic traits.

Second, compared with the United States, Germany is characterized by a lower level of economic inequality (Gini index 2018: 41.4 vs. 31.7; World Bank, 2023). In regions with higher income inequality, social status appears to be more salient (Paskov et al., 2013) and social class effects on psychological outcomes tend to be larger (e.g., Schneider, 2019). Also, social class stereotyping, that is, the perception of higher social classes as more competent compared with lower social classes, increases with higher inequality (Durante et al., 2017). All this considered, social class effects may be particularly pronounced in the United States.

Investigating the effects across a different political and societal context, our studies provide important information on their robustness and generalizability. In Study 1, we relied on self-reports. Participants of a representative sample (*N* = 2239) rated the importance of competence and other attributes in a politician, and also rated how competent they perceived themselves which allowed us to explore the presumed mediation of self-concept.

In Studies 2a/b and 3, we employed a less obtrusive methodology. Going beyond previous studies we did not rely on self-reported trait importance, which may be prone to self-presentation effects. Rather we drew on findings showing an advantage for competent-looking politicians (Todorov et al., 2005). Participants were shown portrait photographs of politicians who varied in their perceived competence according to pretest ratings. For each politician participants indicated their voting likelihood. Using pictures rather than self-reported importance tested whether perceived competence is spontaneously more appealing to voters of higher compared with lower social class.

In all studies, we explore the role of chronic SSS in addition to SES. Study 1 explored whether SSS and SES had similar effects on people’s self-schema and whether possible differences in self-schema might be responsible for the diverging effects of SES and SSS found in previous research by Callaghan et al. (2022).

We had no firm expectation about the importance of warmth in politicians depending on SES or SSS. Assuming that social classes differ in how important they consider certain traits in a politician because of differences in their self-concept, it is not clear what to expect regarding the importance of warmth. Research shows mixed results on the class stereotype on warmth depending on country (Durante et al., 2013, but see also Durante et al., 2017). More crucially, regarding self-concept there was either no (Abele, 2003) or even a weak negative relationship (Boileau, 2022). Finally, there is evidence that individuals with lower SES only prefer warm candidates when their warmth appears to be genuine (Tan & Kraus, 2018).

Two studies were preregistered (Study 2b: https://aspredicted.org/436zf.pdf, Study 3: https://aspredicted.org/s3743.pdf). All preregistrations included study design, stopping rule, exclusion criteria, and planned analyses. All preregistered analyses are reported in the manuscript and any deviations were marked. For each study, sample size was determined before data analysis. All measures, manipulations, and exclusions of the presented studies are reported.

## Study 1

In Study 1, we investigate if people’s social class is related to how important they consider competence in political candidates. Specifically, we rely on self-reported importance ratings which were part of a large-scale survey with a nationally representative German sample that was originally conducted as part of a different, unrelated research project. To isolate the association between social class and the reported importance of competence, we tested this effect relative to the other three main facets of social judgments in the Big Two model (Abele et al., 2016)—assertiveness, warmth and trustworthiness.

Especially separately investigating competence (as ability) and assertiveness (as motivational component) might provide new insights. While most of the previous research in this domain referred to competence but actually measured a combination of both traits (e.g., Callaghan et al., 2022; Funk, 1999), there are first studies indicating that competence and assertiveness should be dissociated when investigating weight attached to candidate traits (Mignon et al., 2016). Consequently, a differentiation between both traits can help to better understand social class effects.

We also assessed participants’ self-schema to explore its role as possible mediator. In addition to investigating the role of SES, we explored whether SSS showed a similar pattern.

Finally, we attempt to rule out individuals’ political orientation as alternative explanation. A higher income has often been linked with a more right political orientation (e.g., Page et al., 2013). In addition, political orientation is associated with the value attached to assertiveness (Eriksson, 2018). Even though competence has often been considered a non-ideological dimension (e.g., Mignon et al., 2016), it is crucial to clarify if political orientation plays a role in the association between social class and the importance of competence in politicians.

### Method

All materials (exceptions for Study 2a/b and 3: politicians’ pictures (shared upon request); materials of the pretest), R code for all reported analyses, data and codebooks are available at https://osf.io/rfvbj/

#### Materials

Participants indicated how important they considered being “competent,” “assertive,” “likeable,” and “trustworthy” for a politician on a scale from 1 (*not at all important*) to 5 (*very important*).

To explore the presumed mediation by self-schema, we assessed participants’ self-perception on these characteristics with three attributes each (Cronbach’s α = .77–.87) on a scale from 1 (*not at all*) to 5 (*completely*) (see Supplemental Tables S1 and S2).

#### Participants

Data from 2469 German participants were collected via an online access panel provider. The sample was representative for the German population regarding age, gender and education. After excluding participants who did not know their annual household income and participants who did not want their answers to be used, we arrived at a final sample of 2239 participants (1095 female, 1136 male, eight diverse, *M*_age_ = 46.02 years, *SD*_age_ = 14.33). This sample size was sensitive to detecting an effect size of *r* = .05 or higher given 80% power and alpha = 0.05 (Faul et al., 2009).

#### Procedure

The questions were part of a larger online study which was conducted with SoSci Survey (Leiner, 2019) and introduced as a study on political attitudes. All variables of interest for Study 1, aside from people’s self-perceived traits, were assessed before an experimental manipulation that was part of a different, unrelated research project. Self-perceived traits did not differ significantly between the experimental and control groups (all *p*s > .200).

First, demographics were assessed (gender, age, federal state). Then, SSS was measured with the MacArthur Scale (e.g., Adler et al., 2000) and participants indicated their educational level on a scale with 8 options plus an “other” option (*Median* = secondary school certificate). Next followed further questions on (political) attitudes that were not relevant to the present paper, as well as one item for global political orientation (1 = *left*; 9 = *right*). Then, participants rated the importance of competence and further candidate traits as described above as well as the importance of further characteristics irrelevant to the present paper (e.g., having a good concept for climate protection). After an experimental manipulation and further variables irrelevant to this paper (see Supplemental Material (1) for a complete list) participants indicated their self-reported competence, assertiveness, warmth and trustworthiness. Finally, they reported their current monthly net household income on category options from 1 (*below 500€*) to 13 (*10,000€ and more*) (*Median = 2,000€–below 2,500€*) plus a “don’t know” option.

### Results

#### Reported Importance of Competence

Overall, participants indicated that competence in a politician was rather important (*M* = 4.39, *SD* = 0.85, 5-point scale). We coded and z-standardized educational level and household income and created an index of SES by taking their mean (see Kraus & Keltner, 2009). Consistent with our hypothesis, a higher SES was significantly positively associated with reporting a higher importance of competence in political candidates, *r*(2237) = .08, 95% CI [0.04, 0.13], *p <* .001.

However, the investigated sample was representative only with regard to education but not income. A comparison of the income distribution in our sample with the German population (German Federal Statistical Office, 2022) shows that our sample is characterized by an overrepresentation of individuals with lower household income and an underrepresentation of individuals from the highest income category of 5000€ and above (see Supplemental Table S3). Thus, we created a subset which was representative for the German population regarding household income. For this purpose, we divided the sample into ten income categories parallel to those described in the Microcensus and randomly drew individuals from these subgroups to obtain a new dataset with the same ratio of income categories as the German population. In this subset (*N* = 1200), again a higher SES was significantly associated with a higher importance of politicians’ competence, *r*(1198) = .10, 95% CI [0.04, 0.15], *p* < .001.

#### Robustness Checks

Overall, SES was not associated with political orientation, *r*(2237) = .01, 95% CI [−0.03, 0.05], *p =* .597. Importantly, the association between SES and the rated importance of competence remained robust when controlling for political orientation, *b* = .09, 95% CI [0.05, 0.14], *SE* = 0.02, *t*(2239) = 4.06, *p* < .001.

As shown in Table 1, SES was not significantly related to the reported importance of assertiveness or warmth, but unexpectedly, people with higher SES indicated a higher importance of trustworthiness. When controlling for the reported importance of the other three facets of the Big Two model (Abele et al., 2016), SES continued to positively predict the importance of competence in politicians, *b* = .06, 95% CI [0.03, 0.09], *SE* = .01, *t*(2234) = 3.92, *p* < .001.

**Table 1.** Descriptive Statistics and Bivariate Correlations Among Objective SES, Subjective Social Status, Political Orientation, the Rated Importance of Candidate Traits and Voters’ Traits (Study 1).

Variables	1	2	3	4	5	6	7	8	9	10	11
1. SES											
2. SSS	.53										
3. Political Orientation	.01	.07									
4. Importance Competence	.08	.07	−.06								
5. Importance Assertiveness	.01	.07	.02	.65							
6. Importance Warmth	−.00	.07	.00	.47	.48						
7. Importance Trustworthiness	.05	.06	−.05	.72	.65	.54					
8. Own Competence	.19	.25	.05	.35	.35	.28	.33				
9. Own Assertiveness	.14	.26	.13	.13	.23	.19	.15	.62			
10. Own Warmth	.01	.10	−.03	.37	.37	.36	.38	.67	.36		
11. Own Trustworthiness	.05	.07	−.04	.43	.39	.32	.42	.68	.34	.83	
*M*	0.00	5.03	5.63	4.39	4.16	3.84	4.29	3.66	3.12	3.86	3.99
*SD*	0.79	1.75	1.86	0.85	0.86	0.96	0.87	0.77	0.85	0.85	0.83

*Note. N* = 2239. Political Orientation was assessed on a scale from 1(*left*) to 9 (*right*). Self-perceived traits were mean scores based on three items per trait. All correlations ≥.05 or ≤−.05 are statistically significant (*p* < .05). SES = socioeconomic status; SSS = subjective social status.

#### SSS

Overall, SSS was positively correlated with SES, *r* (2237) = .53, 95% CI [0.50, 0.56], *p* < .001. Similar to SES, the importance of competence increased with higher SSS, *r*(2237) = .07, 95% CI [0.03, 0.11], *p* < .001. However, SSS was significantly positively correlated with the reported importance of all of the candidate traits (see Table 1). In contrast to SES, SSS was not significantly related to the importance of competence when controlling for the importance of assertiveness, warmth and trustworthiness, *b* = .01, 95% CI [−0.00, 0.02], *SE* = .01, *t*(2234) = 1.23, *p* = .219.

#### Mediation Via Self-Perceived Competence

SES and self-perceived competence correlated positively, *r*(2237) = .19, 95% CI [0.15, 0.23], *p* < .001. A mediation analysis using the R package lavaan (version 0.6-12; Rosseel, 2012) suggests that the effect of SES on the importance of competence was completely mediated via self-perceived competence, *b* = 0.07, *SE* = 0.01, *z* = 8.00, *p* < .001, 95% CI [0.05, 0.09]. The subsample representative for household income showed the same result, *b* = 0.09, *SE* = 0.01, *z* = 6.69, *p* < .001, 95% CI [0.06, 0.11].

Furthermore, SSS was positively associated with the self-schema of all of the four traits (see Table 1).

### Discussion

Supporting our hypothesis and using a representative German sample, we found that the higher the SES, the higher the importance of politicians’ competence. This association was independent of political orientation. Furthermore, the data offer preliminary support for differences in the self-concept as presumed cause for this relationship. More concretely, voters with higher SES perceived themselves as more competent and this self-view completely mediated the relationship between SES and the importance of politicians’ competence. This is in line with previous findings from the United States that higher-class voters perceive a greater interpersonal closeness with competent politicians (Callaghan et al., 2022). However, as all variables were measured our study offers merely correlational evidence. Thus, we cannot rule out that other potential mediators may also play a role.

In addition, we acknowledge that the way we assessed self-concept does not inform us to what extent the traits were part of the spontaneously accessible self-concept (schema) that would guide the perception of others (Fong & Markus, 1982; Markus, 1977). While people of high SES also rated themselves as more assertive than people of low SES there was no difference in the importance of this trait for a politician. It seems plausible that people of higher SES are schematic on competence but not on assertiveness as there are many situations and experiences over a lifetime that form and reinforce their perception of being competent. Moreover, competence is unambiguously considered positive. Experiences of assertiveness are probably much less frequent for many people and assertiveness has also negative connotations.

We had predicted the influence of SES on the importance of politicians’ competence but had no hypothesis regarding assertiveness which we only assessed as a control variable. Our findings suggest that a distinction between competence that is merely related to ability and other aspects of taking effective action (namely assertiveness) as suggested by Abele et al. (2016) seems appropriate when assessing the appeal of politicians.

For SSS we found a different pattern. Whereas the rated importance of competence increased with SSS similarly to SES, this relationship was not robust when controlling for the importance of other traits. Apparently, in contrast to people of high SES, people with high SSS do not specifically value competence but generally value desirable traits in politicians more compared with people with lower SSS. A recent meta-analysis (Tan et al., 2020) suggests that high positive correlations between the SSS-ladder and other measures may reflect a positive response bias, which would explain why in our study the correlations between SSS and own traits were higher than for SES. This would imply that that the higher relevance of competence among people high in SSS does not necessarily reflect similar processes as in people high in SES. We will return to this issue in the “General Discussion.”

Altogether we found clear support for our hypothesis that people of higher SES value competence more in politicians compared with people of lower SES. Yet, one caveat needs to be addressed. We only measured what people explicitly reported to be important traits in politicians. It is an open question whether this would really influence their voting decisions. People may mention competence as an important characteristic because they believe that this is what is expected of rational voters. Even if they indeed consider competence as highly important other characteristics may have a larger influence when it comes to actual voting without voters being consciously aware of it. Thus, in the following studies, we investigated the influence of perceived competence on voting likelihood dependent on social class while varying perceived competence less obtrusively and not asking participants directly about how important they considered this predictor.

## Study 2a and 2b

The work by Todorov and colleagues (2005; see also Ballew & Todorov, 2007; Olivola & Todorov, 2010a, 2010b) shows that competence perceived only by a politician’s looks influences voting decisions. Based on the results of Study 1, we expect this effect to be stronger for voters with higher SES. To test this hypothesis, we presented pictures of (unknown) politicians’ faces with high versus low perceived competence in Study 2a/b and measured voting likelihood. This procedure allowed us to test the influence of perceived competence depending on SES in a rather subtle manner. In addition, we differentiated the effects of perceived competence from warmth. Warmth appears to be an important basis of comparison as it is typically juxtaposed with competence as a main dimension of interpersonal perception (e.g., Fiske et al., 2002) and people tend to make rapid judgments of this trait (e.g., Fiske et al., 2007). Again, we measured SSS and explored if it played the same role as SES.

### Method

#### Materials

In each of the two studies, we used ten portrait photos selected from a sample of 63 male members of the Swiss national parliament. All pictures were taken from the parliament’s website which ensured a uniform portrait style, standard business attire and a similar background. In a pretest, 80 participants rated subsets of portraits regarding the attributes “competent” and “warm” on a scale from 1 (*not at all*) to 11 (*very*) such that each portrait was rated by 20 participants. Our goal was to select one set of pictures with low and one with high perceived competence but to keep warmth at a constant level across the sets. Due to an organizational mistake in Study 2a, there were only four pictures in the condition of relatively low perceived competence (pretest competence: *M* = 5.65, *SD* = 0.56, warmth: *M* = 6.38, *SD* = 1.42) and six in the condition of relatively high perceived competence (pretest competence: *M* = 7.08, *SD* = 0.50, warmth: *M* = 6.25, *SD* = 1.34). Therefore, we added Study 2b which contained two equally large sets (low competence set: pretest competence: *M* = 5.54, *SD* = 0.40, warmth (measured via “warm,” “sincere,” “likable ”): *M* = 5.80, *SD* = 0.75; high competence set: pretest competence: *M* = 7.85, *SD* = 0.27, warmth: *M* = 5.89, *SD* = 0.24). The set in Study 2b contained three photos from Study 2a and seven new ones.

#### Participants

For Study 2a, we collected data from 282 German-speaking participants (with incomplete submissions: 330). For Study 2b, we collected data from 260 German participants on prolific.co (with incomplete submissions: 266). An a-priori power analysis using G*Power for alpha = .05 and a power of 80% to detect a small effect of *f* = 0.10 for the interaction effect of a 2 × 2 mixed ANOVA as a proxy resulted in a sample size of 200. We applied our preregistered exclusion criteria for Study 2b (failing the attention check, familiarity with any of the politicians, missing/invalid values on key variables, being no native German speaker^2^) also to Study 2a and additionally excluded participants who failed a seriousness check or were below 18 years. After exclusions we arrived at a final sample size of *N* = 396 (*N*_2a_ = 195, *N*_2b_ = 201) participants (220 female, 172 male, four diverse; *M*_age_ = 28.32 years, *SD* = 9.93; Study 2a: 138 female, 56 male, one diverse; *M*_age_ = 26.06 years, *SD* = 9.46; Study 2b: 82 female, 116 male, three diverse; *M*_age_ = 30.51 years, *SD* = 9.90).

#### Procedure

The procedures in Study 2a/b were almost identical. In both online studies, participants indicated their interest in politics (1 = *not at all*; 7 = *very much*); their political orientation (1 = *left*; 9 = *right*), their voting regularity (11-point scale with higher values indicating a greater regularity) and whether they had been eligible to vote in the last German federal election. Next, SSS was assessed via the MacArthur scale.

Participants were instructed that they would see 10 pictures of randomly selected male politicians. They viewed the faces in an individualized randomized order. Based on measures of Fiske et al. (2002), participants rated perceived warmth (“warm,” “friendly,” “sincere”; α Study 2a/b = .82/.82) and competence (“competent,” “intelligent,” “ambitious”; α Study 2a/b = .74/.65) for each politician on a scale from 1 (*not at all*) to 5 (*extremely*) as manipulation check (see Supplemental Material for the German version, Supplemental Table S4). After this check, participants indicated how likely they would consider voting for this candidate from 1 (*not at all likely*) to 11 (*very likely*).

Finally, participants completed the measures of SES among further demographics (gender, age, German language skills). SES was assessed via educational level as in Study 1 (Study 2a: *Median* = high school diploma; Study 2b: *Median* = university degree) and current annual household income, on a scale from 1 (*below 15,000€*) to 8 (*over 150,000€*) (plus “don’t know” option in Study 2a); Study 2a: *Median* = 50,001€–75,000€; Study 2b: *Median* = 35,001€–50,000€.

Paralleling Study 1, we created an index of SES. Due to the relatively high proportion of students, we additionally computed an *adjusted index* of SES for students and apprentices based on their parents’ education (mean of father’s and mother’s educational level) and current family household income.

Finally, participants indicated if they recognized any of the politicians so that we could exclude these cases. In Study 2a, they indicated whether they had answered all the questions seriously.

### Results

#### Manipulation Check

In both studies, politicians in the high competence set were perceived as significantly more competent than politicians in the low competence set, Study 2a: *M*_high_ = 3.48, *SD*_high_ = 0.44, *M*_low_ = 2.95, *SD*_low_ = 0.50, *t*(194) = 16.10, *p* < .001, *d* = 1.15, 95% CI for *d* [0.97, 1.33]; Study 2b: *M*_high_ = 3.56, *SD*_1_ = 0.44, *M*_low_ = 3.18, *SD*_2_ = 0.43, *t*(200) = 14.48, *p* < .001, *d* = 1.02, 95% CI for *d* [0.85, 1.19]. Although based on the pretest perceived warmth should not differ between the target politicians, competent-looking politicians were rated as less warm than less competent-looking politicians (Study 2a: *M*_1_ = 3.12, *SD*_1_ = 0.46, *M*_2_ = 3.17, *SD*_2_ = 0.48, *t*(194) = −1.72, *p* = .087, *d* = −0.12, 95% CI for *d* [−0.26, 0.02], Jeffrey–Zellner–Siow (JZS) Prior *BF* = 2.94; Study 2b: *M*_high_ = 2.78, *SD*_high_ = 0.50, *M*_low_ = 3.21, *SD*_low_ = 0.53, *t*(200) = −13.36, *p* < .001, *d* = −0.94, 95% CI for *d* [−1.11, −0.78], JZS Prior BF > 1000).

#### Likelihood of Voting

Due to the parallel design of Study 2a/b and to test our hypotheses with more power, we used a joint analysis including data source (Study 2a or 2b) as additional predictor (see Supplemental Material (3) for separate analyses and the preregistered linear regression analysis). We conducted a multilevel linear regression with maximum likelihood estimates. The joint model specified a within-subject relationship between perceived competence and voting likelihood that we had predicted to be stronger for participants with higher SES. We grand-mean centered SES as level-2-predictor and used person-mean centering for condition as effect-coded level-1-predictor (−1 = low competence, 1 = high competence) (see Enders & Tofighi, 2007). Data source was included grand-mean centered as dummy-coded level-2-predictor (0 = Study 2a, 1 = Study 2b).

The results are displayed in Table 2. Competence condition had a significant effect on voting likelihood, *b* = 0.22, *SE* = 0.03, *t* = 7.12, *p* < .001, 95% CI [0.16, 0.28]), implying that participants indicated on average a higher voting likelihood for politicians from the high compared with the low competence set. Consistent with our hypothesis, this effect was moderated by SES (*b* = 0.12, *SE* = 0.04, *t* = 2.90, *p* = .004, 95% CI [0.04, 0.20]).^3^ The remaining model terms were insignificant (see Table 2, Model 1).

**Table 2.** Parameter Estimates for Multilevel Model of Voting Likelihood (Study 2a and 2b).

Fixed effects	Model 1					Model 2				
	*b*	*SE b*	*t*	*p*	95% CI^a^	*b*	*SE b*	*t*	*p*	95% CI^a^
Intercept	5.46	0.07	76.50	<.001	5.32, 5.60	5.47	0.07	76.35	<.001	5.33, 5.61
Competence—Condition	0.22	0.03	7.12	<.001	0.16, 0.28					
Meas. Warmth						1.51	0.04	39.80	<.001	1.43, 1.58
Meas. Competence						1.32	0.04	30.80	<.001	1.23, 1.40
Obj. SES	0.05	0.10	0.54	.588	−0.14, 0.24	0.05	0.10	0.52	.605	−0.14, 0.24
Source	−0.12	0.14	−0.87	.387	−0.42, 0.17	−0.15	0.14	−1.04	.297	−0.43, 0.13
Meas. Warmth × Source						0.18	0.08	2.40	.017	0.03, 0.33
Meas. Competence × Source						−0.13	0.09	−1.56	.120	−0.30, 0.04
Meas. Warmth × Meas. Competence						0.26	0.04	6.99	<.001	0.19, 0.34
Competence—Condition × Obj. SES	0.12	0.04	2.90	.004	0.04, 0.20					
Meas. Warmth × Obj. SES						−0.03	0.05	−0.61	.543	−0.13, 0.07
Meas. Competence × Obj. SES						0.19	0.06	3.21	.001	0.07, 0.30
Obj. SES × Source						0.16	0.19	0.82	.410	−0.22, 0.54
Meas. Warmth × Obj. SES × Source						−0.10	0.10	−0.94	.349	−0.31, 0.11
Meas. Competence × Obj. SES × Source						0.06	0.12	0.53	.595	−0.17, 0.29
Random effects ([co-]variances)										
Intercept			1.65					1.90		
Competence-Condition			0.00	0.01						
Meas. Competence								0.29	0.27	
Meas. Warmth								0.28	0.04	0.00
Residual			3.74					1.22		

*Note. N* = 396, 10 pictures per Study, 3,960 observations. ICC for Model 1 = .31, ICC for Model 2 = .64. *b* = unstandardized coefficient. Competence-Condition −1 = Low Competence, Competence-Condition 1 = High Competence. Meas. = Measured. Source 0 = Study 2a, Source 1 = Study 2b. CI = confidence interval; SES = socioeconomic status; *SE* = standard error.

a Confidence intervals were computed from the profiled likelihood.

**Figure 1.**
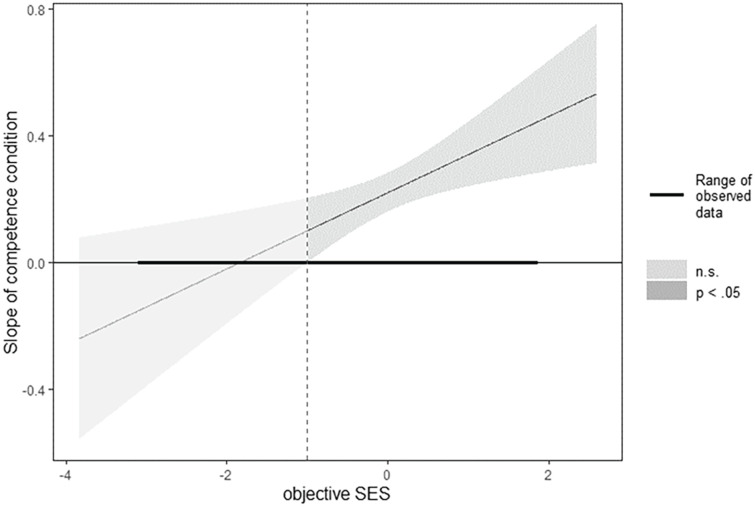
Conditional Effect of Competence Condition on Voting Likelihood as a Function of Objective SES Note. Johnson–Neyman plot for the effect of competence condition on voting likelihood as a function of objective SES. In addition to the point estimate, the 95% Confidence Interval is displayed. The plot was created using the R package interactions (version 1.1.5; Long, 2019). SES = socioeconomic status.

Simple slope analyses revealed that the strongest effect of competence condition on voting likelihood was found among participants with high SES (+1 *SD; b* = 0.31, *SE* = 0.04, *t* = 7.08, *p* < .001, 95% CI [0.23, 0.40]), holding all other covariates constant. At low levels of SES, competence condition still had a positive, but smaller effect (−1 *SD; b* = 0.13, *SE* = 0.04, *t* = 2.99, *p* = .003, 95% CI [0.05, 0.22]). A Johnson–Neyman plot (see Figure 1) shows that the effect of competence condition was significant for all values of SES above −1.00 and increased with SES.

Furthermore, the results of parallel analyses using the adjusted score of SES showed similar significance levels (see Supplemental Table S7).

#### Exploratory Analyses

##### Measured Competence as Predictor

As the manipulation check indicated that perceived warmth and competence of the two sets were confounded, we analyzed the data using measured competence and warmth as predictors. In a parallel analysis, we included both measured traits (from the manipulation check) as level-1-predictors (person-mean centered) with random slopes instead of competence condition. Perceived competence had a significant effect on voting likelihood, *b* = 1.32, *SE* = 0.04, *t* = 30.80, *p* < .001, 95% CI [1.23, 1.40]. In addition, warmth had a significant effect, *b* = 1.51, *SE* = 0.04, *t* = 39.80, *p* < .001, 95% CI [1.43, 1.58]. Importantly, only the interaction effect between competence and SES was significant, *b* = 0.19, *SE* = 0.06, *t* = 3.21, *p* = .001, 95% CI [0.07, 0.30],^4^ but the interaction between warmth and SES was not, *b* = −0.03, *SE* = 0.05, *t* = −0.61, *p* = .543, 95% CI [−0.13, 0.07]. Irrelevant to our hypothesis, the effect of warmth was larger in Study 2b compared with Study 2a, *b* = 0.18, *SE* = 0.08, *t* = 2.40, *p* = .017, 95% CI [0.03, 0.33]. Furthermore, the effect of competence on voting likelihood was larger for politicians with high warmth, *b* = 0.26, *SE* = 0.04, *t* = 6.99, *p* < .001, 95% CI [0.19, 0.34]. The other model terms were nonsignificant (see Table 2, Model 2). Controlling for political orientation, political interest and voting regularity did also not change the pattern of results (*p* < .001). Further robustness checks can be found in the Supplemental Material (Supplemental Tables S8–S11).

##### SSS

When including SSS in the main analyses instead of SES neither the interaction with the competence condition nor the interaction with measured competence on voting likelihood was significant, *p* = .196/*p* = .851 (see Supplemental Table S10).

### Discussion

Whereas Study 1 showed that people of higher SES rated politicians’ competence as more important than people of lower SES, Studies 2a/b showed that this also translates into differences in preference for competent-looking politicians between voters of higher and lower SES. Replicating the results of previous studies (Ballew & Todorov, 2007; Todorov et al., 2005), participants overall indicated a higher voting likelihood for more (vs. less) competent-looking politicians. As expected, the effect of perceived competence on voting likelihood was larger among participants with higher SES. The interaction effect was small, but the result supports our hypothesis that politicians’ perceived competence is more appealing to higher- than to lower-class voters.

Consistent with the finding from Study 1 that the relationship between SSS and the importance of candidates’ competence is less robust, SSS did not play a role in the weighting of perceived competence. Apparently, voters with high SSS explicitly express a higher preference for competent politicians but do not spontaneously take this cue into account. We will address this finding together with the results of SSS from Studies 1 and 3 in the “General Discussion.”

Finally, we must consider that the procedure of assessing perceived competence (and warmth) before voting likelihood may have increased its accessibility. Although this would not explain why the effect was stronger for voters with high SES we cannot claim that people of higher SES would also spontaneously give more weight to competence than people with lower SES. To explore this issue we modified our design for Study 3.

## Study 3

In Study 3, we changed the design of Studies 2a/b regarding several aspects. First, we assessed voting likelihood before warmth and competence. Second, because the dichotomization of faces with low vs. high perceived competence reduces the power, we included a larger sample of pictures in Study 3. Thereby, we ensured that politicians with perceived competence across the whole spectrum of our pretest ratings were included. Third, we replaced the attribute “ambitious” with “capable” to assess perceived competence without any aspects linked to assertiveness.

Finally, we cannot rule out that the effects observed in Studies 2a/b were due to another trait perceived from the faces that was confounded with competence. Of course, we cannot control for all possible traits but chose to control for perceived dominance in Study 3. Dominance plays an important role to achieve social rank aside from demonstrating competence (Cheng et al., 2013). In addition, more dominant-looking people are perceived as having a higher status (Rahal et al., 2021). Thus, a more dominant-looking politician might also elicit a higher perceived similarity among higher-class voters. In Study 3, we therefore controlled for perceived dominance with separately assessed ratings of the politicians’ perceived dominance.

### Method

#### Materials

We used pictures from the same database of politicians as in Studies 2a/b but selected 32 pictures covering the whole range of competence levels based on the pretest. More concretely, we ordered the pictures according to pretest competence ratings and selected every other picture (range on 11-point scale: 5.05–8.5). In addition, we standardized the background to be completely white for all portraits.

In a further study (*N* = 96; 32 female, 63 male, one diverse; *M*_age_ = 31.57, *SD*_age_ = 10.04) run via prolific.co, participants rated the 32 pictures on either how “competent” (*n* = 30), “warm” (*n* = 35), or “dominant” (*n* = 31) they perceived each politician on a scale from 1 (*not at all*) to 5 (*very*).

#### Participants

We conducted a simulation using the R package SIMR (version 1.0.6; Green & MacLeod, 2016) to estimate the power for a cross-level interaction effect in a two-level model (Arend & Schäfer, 2019). Results indicated that 370 participants would suffice to provide 80% power to obtain a standardized effect size of 0.10 keeping alpha at 5%. We preregistered a minimum sample size of 400 valid cases and collected complete data^5^ from 425 German participants on prolific.co. Following preregistered exclusion criteria (failing any attention check, recognizing any of the depicted politicians, missing/invalid values on key variables), we arrived at a final sample of 400 participants (169 female, 220 male, 11 no answer provided; *M*_age_ = 30.59 years, *SD*_age_ = 8.83).

#### Procedure

Study 3 was conducted online. The beginning of the study and the assessment of covariates was identical to Study 2a/b. Then participants read that they would see 32 pictures of male politicians. In the first phase, they viewed the faces individually and indicated their voting likelihood for each as in Study 2a/b. Thereafter, half of the participants (*n* = 203) rated first perceived competence for all politicians and then warmth (Phases 2 and 3) whereas for the others (*n* = 197) the order was reversed. In each study phase, pictures were presented in a randomized order. Perceived competence and warmth were rated as in Study 2a/b except that the attribute “ambitious” was replaced by “capable.”

Based on these measures, we created two scores for each politician: Competence (Cronbach’s α: .81–.89) and warmth (Cronbach’s α: .78–.87).

Afterward, participants completed the same measures of SES including educational attainment (*Median* = university degree) and household income (*Median* = 35,001€–50,000€) as in Study 2a/b among further demographics. Finally, they indicated if they had recognized any politician.

### Results

#### Likelihood of Voting

Again, we computed a composite score of SES. We then computed multilevel linear regression models for voting likelihood as criterion parallel to Study 2a/b. In Model 1, we included measured competence (level 1), SES (level 2) and their interaction. In Model 2, we additionally controlled for warmth (level 1) and its interaction with SES. All level-1-variables were person-mean centered and SES was grand-mean centered. We included a random intercept for participants, random slopes for the level-1-predictors and grouped the data by participants.

Again, measured competence significantly predicted voting likelihood (Model 1), *b* = 1.10, *SE* = 0.03, *t* = 36.61, *p* < .001, 95% CI [1.04, 1.16]. Furthermore, warmth and competence were significantly correlated, *repeated-measures r*(12399) = .35, *p* < .001. Therefore, we controlled for the influence of warmth and its interaction with SES in Model 2. In this model, both competence, *b* = 0.83, *SE* = 0.03, *t* = 28.43, *p* < .001, 95% CI [0.77, 0.88], and warmth, *b* = 0.72, *SE* = 0.03, *t* = 25.30, *p* < .001, 95% CI [0.67, 0.78], significantly predicted voting likelihood. Although the interaction of competence and SES showed the same trend as in the previous studies when controlling for the interaction between warmth and SES, it fails to reach significance at conventional significance levels (Model 2: *b* = 0.08, *SE* = 0.04, *t* = 1.95, *p* = .052, 95% CI [−0.00, 0.15]). The rest of the model terms were nonsignificant (see Table 3).

**Table 3.** Parameter Estimates for Multilevel Models of Voting Likelihood (Study 3).

Fixed effects	Model 1					Model 2				
	*b*	*SE b*	*t*	*p*	95% CI^a^	*B*	*SE b*	*t*	*P*	95% CI^a^
Intercept	5.12	0.06	79.22	<.001	4.99, 5.25	5.12	0.06	79.22	<.001	4.99, 5.25
Warmth						0.72	0.03	25.30	<.001	0.67, 0.78
Competence	1.10	0.03	36.61	<.001	1.04, 1.16	0.83	0.03	28.43	<.001	0.77, 0.88
Obj. SES	0.12	0.08	1.40	.162	−0.05, 0.28	0.12	0.08	1.40	.162	−0.05, 0.28
Warmth × Obj. SES						−0.02	0.04	−0.41	.681	−0.09, 0.06
Competence × Obj. SES	0.03	0.04	0.86	.393	−0.04, 0.11	0.08	0.04	1.95	.052	−0.00, 0.15
Random effects ([co-]variances)										
Intercept			1.57					1.59		
Warmth								0.14		
Competence			0.16	0.29				0.14		
Residual			3.06					2.69		

*Note. N* = 400, 32 pictures, 12,800 observations. Warmth and competence refer to the perceived traits as measured in Study 3. *b* = unstandardized coefficient; SE = standard error; CI = confidence interval; SES = socioeconomic status.

a Confidence intervals were computed from the profiled likelihood.

Including political orientation, political interest and voting regularity as covariates did also not change the pattern and the results were robust to using the adjusted SES score (see Supplemental Material for further robustness checks, Supplemental Tables S14 and S15).

#### Exploratory Analyses

##### Further Independent Ratings of Competence, Warmth, and Dominance

To disentangle perceived competence from dominance as possibly confounded trait, we had an independent sample rate the faces for competence, dominance, and warmth as described above. We repeated the main analyses using these independent competence ratings as predictor (level 2, grand-mean centered) as well as the interaction with SES to predict voting likelihood. In contrast to the competence ratings from Study 3, politicians’ independently perceived competence did not only predict voting for them, *b* = 1.65, *SE* = 0.05, *t* = 33.26, *p* < .001, 95% CI [1.55, 1.74]), but—in line with our hypothesis—this effect was significantly moderated by SES, *b* = 0.15, *SE* = 0.06, *t* = 2.36, *p* = .018, 95% CI [0.03, 0.28]).

In addition including independent dominance and warmth ratings showed that the interaction between competence and SES remained robust, *b* = 0.18, *SE* = 0.06, *t* = 2.78, *p* = .005, 95% CI [0.05, 0.31]). Furthermore, SES significantly moderated the effect of dominance, *b* = 0.13, *SE* = 0.05, *t* = 2.50, *p* = .013, 95% CI [0.03, 0.23]). Higher perceived dominance was significantly associated with *lower* voting likelihood at low levels of SES but not at higher levels (see Supplemental Table S16 in the Supplemental Material for complete results).

##### Subjective Social Status (SSS)

When including SSS in the main analysis (Model 2) instead of SES, it did not significantly interact with measured competence, *p* = .082.

### Discussion

Study 3 replicated the finding from Study 2a/b with more fine-grained stimulus material insofar as looking competent as well as looking warm increased a politician’s electoral success. This appeared to be the case even when these dimensions were not made salient.

Regarding our main hypothesis that specifically perceived competence is more important to voters of higher SES, the results are in line with the predictions and our previous studies, but the effect was small and depending on the analysis was or was not significant. The effect fell beneath conventional significance levels (*p* < .052) when using the competence ratings assessed in the same study as voting likelihood, it was significant when using competence ratings from an independent study (*p* < .018). Moreover, an analysis of extreme cases, similar to Studies 2a/b (see Supplemental Material (6)), also supported the hypothesis. Given that all analyses pointed in the same direction we interpret the data as supporting our hypothesis and previous studies.

Study 3 ruled out that perceived dominance may have caused the effect. Controlling for dominance strengthened rather than weakened the effect. The independence of competence and dominance is in line with the results of Study 1 which found class effects on the weighting of competence but not of assertiveness and again speaks for separating the subfacets of agency. Interestingly, we find first evidence that dominance can lead to negative evaluations of politicians among lower-class voters while it apparently does not matter to higher-class voters. However, this finding is preliminary, and its deeper discussion exceeds the scope of this paper.

Again, replicating the finding of Study 2a/b, the results for SSS did not parallel those of SES. We discuss the apparent divergent patterns for SES and SSS in the “General Discussion.”

## Mini Meta-Analysis

Overall, Studies 2a/b and 3 point in the same direction that voters’ social class moderates the effect of perceived competence from politicians’ faces on voting likelihood. However, the effect sizes were small and depending on study and analysis not always significant (Study 3, *p* = .052). Thus, we conducted a small-scale fixed-effects meta-analysis (Goh et al., 2016) for Studies 2a, 2b, and 3 (included separately, see Supplemental Material (8)). For the interaction effect between SES and perceived competence when controlling for warmth and its interaction with SES, the mini meta-analysis showed a significant effect, *M r* =.14, 95% CI [0.07, 0.20], *Z* = 3.81, *p* < .001, attesting that a politician’s competent appearance influenced voters of high SES more than those of low SES.

## General Discussion

Four studies extend previous work on the role of perceived competence when evaluating politicians by showing that the preference for competence depends on voters’ social class. More concretely, perceived competence appears to be more important for higher than for lower class voters. In Study 1, voters of higher SES rated competence in a politician as more important compared with voters with of SES. This result complements previous findings (Callaghan et al., 2022; Laustsen & Bor, 2017). Observing this effect in the German context which is characterized by a lower economic inequality and thus potentially smaller social class effects compared with the United States speaks to the robustness of a link between social class and a preference for competence. Moreover, the effect does not seem to be limited to an extremely individualistic society as the United States.

Whereas Study 1 provides evidence from a representative sample based on explicit ratings, Studies 2 and 3 go one step further and use a more implicit method by assessing the voting likelihood for politicians varying in competent appearance based on looks. According to the results, politicians’ perceived competence has a larger influence on voting likelihood among higher-class voters. This was the case even when controlling for other impressions elicited by the faces, namely warmth and dominance. Thus, our research provides first evidence that social classes do not only differ in what they say they find important in a politician but also in what they are actually considering, that is, less controlled responses.

A potential mechanism underlying the preference for competence among higher-class voters is voters’ self-perception. We argue that high competence is more likely part of higher (vs. lower) social class people’s self-schema. Given that the self-schema guides the perception and evaluation of others (Carpenter, 1988; Fong & Markus, 1982; Green & Sedikides, 2001; Riggs & Cantor, 1984) people of higher social class should perceive and judge others according to competence cues. Moreover, people generally prefer politicians who are similar to them (Caprara et al., 2007). Study 1 supported our assumptions. Not only did participants of higher SES perceive themselves as more competent, but this self-concept fully mediated the effect of SES on the weighting of competence. Furthermore, exploratory analyses for Studies 2a/b and 3 (see Supplemental Material (5) for details) indicated that people of higher SES did not only place a larger weight on perceived competence but, independent of this preference, also differentiated more between politicians with high and low competence than participants of lower SES. The finding that higher-class voters perceived politicians’ competence in a more nuanced way than lower-class voters suggests that they are more sensitive for competence-related cues in politicians’ appearance. This supports the assumption of higher-class people being competence schematics which makes them attend more to this trait when judging others (Fong & Markus, 1982).

A comparison of Studies 2a/b and 3 suggests that the effect appears to be stronger when competence is made salient beforehand. Apparently drawing perceivers’ attention to competence has different effects depending on social class. We acknowledge that further evidence is needed. But even if the effects were limited to prior activation of competence as in Studies 2a/b this may be closer to real world situations than the absence of prior activation. It seems unlikely that voters form their first impression of a candidate in the voting booth. Rather following a candidate’s public appearances and media coverage makes it likely that an impression of competence is explicitly activated. Accordingly, the increased accessibility of competence elicited by the question order in Study 2a/b might resemble the actual psychological processes when evaluating politicians.

Whereas we found converging evidence for the effect of SES the results do not support similar conclusions for SSS. When explicitly asked (Study 1) high SSS increased the relevance of competence but also of all other traits and the self-ratings on these traits rendering inflated effects due to common method variance likely (see Tan et al., 2020). This would imply that the higher relevance of competence among people high in SSS is an artifact and does not reflect similar processes as in people high in SES. Likewise, the lack of effects of SSS on the more subtle measure of Studies 2a–3 speaks against the functional equivalence of SSS and SES in this regard. This is also in line with previous research that did not find effects of manipulated SSS on the relevance of politicians’ competence (Callaghan et al., 2022). Our assumption of people of high SES being competence schematics was based on the fact that in their environment that is characterized by educational and financial achievement competence plays an omnipresent role and becomes important when judging the self as well as others. To the extent that SSS represents similar socialization processes its effects should be similar. However, SSS is more dependent on the current context in which it is assessed (Destin et al., 2017) and potentially less closely linked with socialization processes. Future research may shed light on the divergent findings on SES and SSS.

Our main result has implications for the well-established effect of perceived competence on electoral outcomes (e.g., Todorov et al., 2005). Generally, members of higher social class show a higher level of political participation (e.g., Kraus et al., 2015). Also, in our studies higher class participants were more likely to have voted in the last German federal election (Study 1: *r* = .22, *p* < .001) and reported to vote more regularly (Study 2a/b: *r* = .18, *p* < .001, Study 3: *r* = .19, *p* < .001). Hence, higher class voters might partially drive the effect of perceived competence on electoral success through their relatively higher turnout rate. For political actors, it appears worthwhile to consider the social class of the target audience in the campaign. Focusing on competence may be a promising strategy for winning voters of higher social class but may not pay off among voters of lower social class.

As our research focused on competence the stimulus material maximized variance on competence and any conclusions regarding other traits can only be preliminary. With this in mind, we summarize that in none of the studies did we observe a class difference on the importance of warmth. Although research reports a warmth stereotype for lower social status groups (Durante et al., 2017), based on which one may expect that warmth is more important to voters of lower social class, it should be noted that this stereotype is not observed in all countries (Durante et al., 2013). More crucially, our assumptions are based on self-stereotypes, and we did not find that people of lower SES think of themselves as warmer than people of high SES.

### Limitations and Future Directions

As voting plays an essential role for the functioning of democracies, it is important to examine which factors influence voting decisions. The current studies add to research on how interpersonal perception influences electoral outcomes. However, we acknowledge some limitations.

First, the characteristics of the politicians’ faces that were used in Studies 2a–3 limit the generalizability of our results. Specifically, we used pictures of white,^6^ male politicians. Specifically, the weight attached to perceived competence might be different for male and female politicians. It has been shown that competence-related information is more important when evaluating female candidates (Ditonto, 2017). Also, none of the candidates depicted was characterized by an extreme lack of perceived competence. This might be due to the fact that these politicians were part of the Swiss parliament and, hence, already had had some electoral success. Future research should investigate the role of social class for weighting competence when evaluating politicians of different gender and ethnic background as well as politicians with a greater lack of perceived competence.

Second, the samples in Studies 2a–3 were not representative of all voters as they did not include people from the extreme ends of the social class spectrum. Especially people with low education did not participate in these studies. This, however, suggests that the weighting of perceived competence which we found despite the restricted variance in SES might be even larger in real-world settings.

Third, the societal and political context of our studies always poses a limitation to our research. Investigating social class effects with German samples supports the generalizability of previously found effects in the United States context, but still more studies with samples from different societies are needed to generalize the results. In fact, assuming that competence is more valued in individualistic than collectivistic societies and class differences manifest more on traits generally regarded highly in a society (Gobel & Miyamoto, 2022) one may expect a divergent pattern for collectivistic societies.

As a further issue, as we cannot manipulate SES we cannot determine whether the observed effect is not due to a third variable. Nevertheless, our hypothesis was based on assumptions about differences in the self-concept between people of higher and lower SES and we found evidence for this assumed mediating process.

### Conclusion

The present research underlines the importance for politicians of being perceived as competent. But perceived competence—even when only based on facial appearance—appears more important for voters of higher compared with lower social class. The reason for this difference seems to lie in systematic differences in the self-concept of members of higher and lower class. Generally, social class has only recently been discovered as predictor for fundamental psychological processes (e.g., Kraus et al., 2012) like person perception. As social class has an omnipresent influence on a person’s identity it is high time psychological research pays more attention to its impact.

## Supplemental Material

sj-docx-1-psp-10.1177_01461672231181465 – Supplemental material for Looking Competent Does Not Appeal to All Voters Equally: The Role of Social Class and Politicians’ Facial Appearance for Voting Likelihood Supplemental material, sj-docx-1-psp-10.1177_01461672231181465 for Looking Competent Does Not Appeal to All Voters Equally: The Role of Social Class and Politicians’ Facial Appearance for Voting Likelihood by Fabienne Unkelbach, Tatjana Brütting, Nina Schilling and Michaela Wänke in Personality and Social Psychology Bulletin

## Notes

1. The authors do not interpret this effect.

2. Even though we had preregistered for Study 2b to exclude non-native German speakers, we did not apply this strict criterium to Study 2a. Here, we included seven participants who reported to speak German for more than 10 years. Excluding these did not change the results.

3. Separate analyses showed that the interaction effect was only significant in Study 2a, *p* = .021, and not in Study 2b, *p* = .224. In Study 2b, the interaction only reached significance for the adjusted score, *p* = .017. Hence, we conducted a meta-analysis across Study 2a, 2b, and 3 (see below).

4. This was also the case for 2b, *p* = .007, while the effect was marginally significant in Study 2a, *p* = .057.

5. Among the incomplete participations were 79 participants who failed an attention check and 23 participants who did not complete the questionnaire.

6. As are over 99% of the members of the German parliament (as of 2023).
