# Supplementary material for: Looking Competent Does Not Appeal to All Voters Equally: The Role of Social Class and Politicians’ Facial Appearance for Voting Likelihood
Source: Pers Soc Psychol Bull. 2023 Jul 7;51(1):66–80. doi: 10.1177/01461672231181465 (PMC11616214; doi:10.1177/01461672231181465)
Supplement: sj-docx-1-psp-10.1177_01461672231181465 – Supplemental material for Looking Competent Does Not Appeal to All Voters Equally: The Role of Social Class and Politicians’ Facial Appearance for Voting Likelihood [file sj-docx-1-psp-10.1177_01461672231181465.docx]

**Online Supplement**

1. **Study 1: Description of Measures and Details on the Manipulation**

Variables were assessed in the following order: Demographics (gender, age, federal state), SSS (MacArthur Scale; Adler et al., 2000), educational attainment on a scale with 8 options plus an “other” option, general political interest, a short scale on German satisfaction with the political system (SPS; Dentler et al., 2020), trust in institutions (adapted from GLES, 2022), specific political attitudes, a short scale on political interest (PIKS; Otto & Bacherle, 2011) a short scale on political efficacy (PEKS; Beierlein et al., 2012), political orientation, party identification, strength of party identification, voting preference for candidate and party, voting turnout in the last German federal election.

Then, relevant to our research question, participants rated the importance of competence, assertiveness, warmth and trustworthiness as well as the importance of the following further candidate traits: A concern for the well-being of fellow human beings, the position within one’s party/politics (being completely in line with the party, having a strong position within the party, being able to balance different interests), having control over his private life and having a good concept for climate protection/stimulating the economy again/combating social inequality/Germany’s foreign policy/strengthening internal security.

Next, they indicated their need for cognition (NFC-K; Beißert et al., 2015), two items on political knowledge as well as an attention check item.

**Part one of the information selection task**

Next, participants completed an information selection task which included an experimental manipulation that was part of a different, unrelated research project: Firstly, to get acquainted with the task, participants had the chance to select some information on two male, fictional political candidates. It was randomized to which of the six largest existing German parties these two candidates ostensibly belonged and made sure that the two politicians in a pair did not belong to the same party. Due to all possible party pairings, participants were randomly split in 15 different settings. The information presented was identical for control and experimental groups and did not contain any contradictions with the substantive positions of the politicians’ respective parties. For each of the two candidates two headlines were presented and participants could click on them to receive the regarding information (e.g.: the headline: “[Candidate 1]’s position on energy policy” lead to the information “[Candidate 1] supports phasing out coal by 2030 at the latest”. Then, participants had to indicate their impression of the candidates based on the selected information on a feeling thermometer from 0 (*negative*) to 100 (*positive*) for each of the candidates. Additionally, they rated the candidate-party fit for both candidates on a scale from 1 (*not at all*) to 5 (*very well*).

**Part two of the information selection task – experimental manipulation**

Then followed the main information selection task. Participants were randomly assigned to 15 experimental (*n* = 1800) and 15 control groups (*n* = 439) by a quota of 4:1 which differed in the types of information presented about the two politicians. Again, participants saw the names of the same two candidates as in the warm-up task with their party membership side by side and were presented with eight headlines per politician on which they could click to receive further information on the candidate. Thereby, based on pretest ratings, information type was manipulated on the congruence with party positions (contradictory vs. non contradictory) and on the relevance for the political duties (role-related vs. non-role-related): Specifically, half of the information in the experimental group appeared to contradict the substantive positions of the politicians’ respective parties and the other half of the information that was non-contradictory. Additionally, half of the information was role-related and the other half of the information was non role-related (crossed with contradictory and non-contradictory information).

All information in the control group was non-contradictory and half of them was role-related whereas the other half was non-role-related.

**Further Measures**

After the manipulation, participants rated the fictional candidates on several measures in the following order: The impression of each candidate on a feeling thermometer (see above), each candidate’s perceived agency and communion on the same scale as used for participants’ self-perceived traits, the perceived candidate-party fit on the same item as mentioned above, each candidate’s perceived political orientation on a scale from 1 (*left*) to 11 (*right*), the impression of the candidate regarding the attributes for which participants had indicated their importance beforehand, the assumed positions of the candidates’ attitude towards redistribution/immigration/combatting climate change, the voting intention for each candidate and his ostensible party.

Finally, participants completed a political knowledge quiz consisting of 10 items adapted from GESIS (2019) and indicated their confidence in their political knowledge. Then, they were asked to recall the information they had read on the candidates, indicated again the perceived candidate-party fit, the perceived attributes mentioned above and their voting intention for candidate and party.

Then followed the assessment of participants’ self-perceived competence (Cronbach’s α = .86), assertiveness (Cronbach’s α = .77), warmth (Cronbach’s α = .87), and trustworthiness with three items per trait (Cronbach’s α = .87; see Tables S1 and S2 below) on a scale from 1 (not at all) to 5 (completely), a 5-item scale on the perceived difficulty of the above-mentioned information selection task, a measure of the current monthly net household income and several items to assess the data quality (consent for data usage, meaningfulness of responses, extent of distraction, open comments) before participants were debriefed.

The exact wording of all variables can be found in the Codebook for Study 1 as well as in the material for Study 1 in the osf-project for this paper.

**Table S1**

*German Original Version of Items Used to Measure Participants’ Self-Perceived Competence, Assertiveness, Warmth and Tustworthiness, Study 1*

| Agency | |  | Communion | |
| --- | --- | --- | --- | --- |
| Assertiveness | Competence |  | Warmth | Trustworthiness |
| Selbstsicher | Kompetent |  | Warmherzig | Vertrauenswürdig |
| Durchsetzungsfähig | Intelligent |  | Sympathisch | Rücksichtsvoll |
| Dominant | Fähig |  | Freundlich | Aufrichtig |

**Table S2**

*English Translation of Items Used to Measure Participants’ Self-Perceived Competence, Assertiveness, Warmth and Tustworthiness, Study 1*

| Agency | |  | Communion | |
| --- | --- | --- | --- | --- |
| Assertiveness | Competence |  | Warmth | Trustworthiness |
| Confident | Competent |  | Warm-hearted | Trustworthy |
| Assertive | Intelligent |  | Likeable | Considerate |
| Dominant | Capable |  | Friendly | Sincere |

**Table S3**

*Comparison of the Proportion of Monthly Household Net Income Categories in Study 1 versus German Microcensus 2021*

| Microcensus 2021 | | |  | Study 1 | | | | | |
| --- | --- | --- | --- | --- | --- | --- | --- | --- | --- |
| Categories | Proportion in % | |  | Categories | | | Proportion in % | |  |
| below 500€ | | 1.9 | | |  | below 500€ | | 3.8 | |
| 500€ - below 1000€ | | 7.5 | | |  | 500€ - below 750€ 750€ - below 1000€ | | (3.4 + 5.9)  9.3 | |
| 1000€ - below 1250€ | | 5.9 | | |  | 1000€ - below 1250€ | | 6.1 | |
| 1250€ - below 1500€ | | 5.9 | | |  | 1250€ - below 1500€ | | 6.3 | |
| 1500€ - below 2000€ | | 13.7 | | |  | 1500€ - below 2000€ | | 13.4 | |
| 2000€ - below 2500€ | | 13.2 | | |  | 2000€ - below 2500€ | | 13.0 | |
| 2500€ - below 3000€ | | 10.2 | | |  | 2500€ - below 3000€ | | 12.5 | |
| 3000€ - below 3500€ 3500€ - below 4000€ | | (8.9 + 7.3)  16.2 | | |  | 3000€ - below 4000€ | | 17.3 | |
| 4000€ - below 5000€ | | 10.4 | | |  | 4000€ - below 5000€ | | 10.5 | |
| 5000€ and above | | 14.5 | | |  | 5000€ - below 7500€ 7500€ - below 10000€ 10000€ and above | | (6.1 + 0.9 + 0.8)  7.8 | |
| *NA* | | 0.6 | | |  | *NA* | | - | |
| *Note. N Study 1* = 2239. Microcensus data obtained from the German Federal Statistical Office (Destatis) (2022). | | | | | | | | | |

**Robustness Check for Study 1: Results of Main Analyses with Reduced Sample Based on Quality Checks**

As a robustness check, we additionally excluded participants who indicated that they had given some meaningless answers or had one or more distractions during the answering process. This resulted in a reduced sample of 1721 participants. Importantly, SES was still significantly related with the reported importance of politicians’ competence, *r*(1719) = .08, 95% CI [0.04, 0.13], *p* < .001 and with self-perceived competence, *r*(1719) = .19, 95% CI [0.14, 0.23], *p* <.001. Additionally, the effect of SES on the importance of competence was still completely mediated via self-perceived competence, *b* = 0.07, *SE* = 0.01, *z* = 7.02, *p* < .001, 95% CI [0.05, 0.09].

Again, people with higher SSS indicated a higher importance of politicians’ competence *r*(1719) = .07, 95% CI [0.02, 0.12], *p* = .004 and a higher self-perceived competence, *r*(1719) = .24, 95% CI [0.20, 0.29], *p* <.001. Nevertheless, similar to the results in the complete sample, merely SES significantly predicted the importance of competence when controlling for the importance of assertiveness, warmth and trustworthiness, *b* = 0.05, *SE* = 0.02, *t*(1716) = 3.27, *p* = .001 (for SSS: *b* = 0.01, *SE* = 0.01, *t*(1716) = 1.09, *p* = .276).

1. **Assessment of Politician’s Perceived Competence and Warmth in Study 2a/b - 3**

**Table S4**

*German Original Version of Items Used to Measure Politicians’ Perceived Competence and Warmth in Study 2a/b and 3*

| Study Number | Competence | Warmth |
| --- | --- | --- |
| 2a/b | Kompetent, Intelligent, Ehreizig | Warm, Freundlich, Aufrichtig |
| 3 | Kompetent, Intelligent, Fähig | Warm, Freundlich, Aufrichtig |

1. **Separate Analyses for Study 2a and 2b**

**Table S5**

*Parameter Estimates for Multilevel Models of Voting Likelihood* ***for Study 2a***

|  | Model 1 | | | | | | | | |  | Model 2 | | | | | | | |
| --- | --- | --- | --- | --- | --- | --- | --- | --- | --- | --- | --- | --- | --- | --- | --- | --- | --- | --- |
| Fixed effects | *b* | *SE b* | | *t* | | *p* | | *95% CI^a^* | |  | | *b* | *SE b* | | *t* | | *p* | *95% CI^a^* |
| Intercept | 5.55 | 0.10 | | 57.86 | | <.001 | | 5.36, 5.74 | |  | | 5.55 | 0.10 | | 57.74 | | <.001 | 5.36, 5.74 |
| Competence – Condition | 0.44 | 0.05 | | 9.41 | | <.001 | | 0.35, 0.53 | |  | |  |  | |  | |  |  |
| Meas. Warmth |  |  | |  | |  | |  | |  | | 1.41 | 0.05 | | 27.20 | | <.001 | 1.31, 1.52 |
| Meas. Competence |  |  | |  | |  | |  | |  | | 1.39 | 0.06 | | 24.19 | | <.001 | 1.27, 1.50 |
| Obj. SES | -0.05 | 0.13 | | -0.42 | | .678 | | -0.31, 0.20 | |  | | -0.04 | 0.13 | | -0.27 | | .785 | -0.29, 0.22 |
| Meas. Warmth x  Meas. Competence |  |  | |  | |  | |  | |  | | 0.19 | 0.05 | | 4.05 | | <.001 | 0.10, 0.29 |
| Competence – Condition x  Obj. SES | 0.14 | 0.06 | | 2.30 | | .021 | | 0.02, 0.27 | |  | |  |  | |  | |  |  |
| Meas. Warmth x Obj. SES |  |  | |  | |  | |  | |  | | 0.02 | 0.07 | | 0.30 | | .766 | -0.12, 0.17 |
| Meas. Competence x  Obj. SES |  |  | |  | |  | |  | |  | | 0.15 | 0.08 | | 1.92 | | .057 | -0.00, 0.31 |
|  | | |  | |  | |  | |  |  | |  | |  | |  |  |  |

**Table S5 (continued)**

| Random effects | |  |  |  |  |  |  |  |  |  |  |
| --- | --- | --- | --- | --- | --- | --- | --- | --- | --- | --- | --- |
| Intercept |  |  | 1.39 |  |  |  |  |  | 1.67 |  |  |
| Competence-Condition |  |  | 0.00 | 1.00 |  |  |  |  |  |  |  |
| Meas. Competence |  |  |  |  |  |  |  |  | 0.28 | 0.24 |  |
| Meas. Warmth |  |  |  |  |  |  |  |  | 0.26 | 0.06 | -0.28 |
| Residual |  |  | 4.04 |  |  |  |  |  | 1.27 |  |  |

*Note.* *N* = 195, 10 pictures per Study, 1,950 observations. ICC for model 2 = .61. *b* = unstandardized coefficient, *SE* = standard error.
Competence-Condition -1 = Low Competence, Competence-Condition 1 = High Competence, Meas. = Measured.
^a^Confidence intervals were computed from the profiled likelihood.

**Table S6**

*Parameter Estimates for Multilevel Models of Voting Likelihood* ***for Study 2b***

|  | Model 1 | | | | |  | Model 2 | | | | | |
| --- | --- | --- | --- | --- | --- | --- | --- | --- | --- | --- | --- | --- |
| Fixed effects | *b* | *SE b* | *t* | *p* | *95% CI^a^* |  | | *b* | *SE b* | *t* | *p* | *95% CI^a^* |
| Intercept | 5.38 | 0.11 | 51.14 | <.001 | 5.17, 5.59 |  | | 5.39 | 0.11 | 51.17 | <.001 | 5.19, 5.60 |
| Competence – Condition | 0.02 | 0.04 | 0.54 | .593 | -0.06, 0.10 |  | |  |  |  |  |  |
| Meas. Warmth |  |  |  |  |  |  | | 1.60 | 0.06 | 28.61 | <.001 | 1.49, 1.71 |
| Meas. Competence |  |  |  |  |  |  | | 1.24 | 0.06 | 19.70 | <.001 | 1.12, 1.37 |
| Obj. SES | 0.14 | 0.14 | 1.02 | .307 | -0.13, 0.40 |  | | 0.14 | 0.14 | 1.02 | .307 | -0.13, 0.41 |
| Meas. Warmth x  Meas. Competence |  |  |  |  |  |  | | 0.38 | 0.06 | 6.22 | <.001 | 0.26, 0.50 |
| Competence – Condition x  Obj. SES | 0.06 | 0.05 | 1.22 | .224 | -0.04, 0.17 |  | |  |  |  |  |  |
| Meas. Warmth x Obj. SES |  |  |  |  |  |  | | -0.08 | 0.07 | -1.41 | .254 | -0.23, 0.06 |
| Meas. Competence x  Obj. SES |  |  |  |  |  |  | | 0.22 | 0.08 | 2.71 | .007 | 0.05, 0.39 |

**Table S6 (continued)**

| Random effects | |  |  |  |  |  |  |  |  |  |  |
| --- | --- | --- | --- | --- | --- | --- | --- | --- | --- | --- | --- |
| Intercept |  |  | 1.89 |  |  |  |  |  | 2.12 |  |  |
| Competence-Condition |  |  | 0.00 |  | 1.00 |  |  |  |  |  |  |
| Meas. Competence |  |  |  |  |  |  |  |  | 0.31 | 0.29 |  |
| Meas. Warmth |  |  |  |  |  |  |  |  | 0.33 | 0.02 | 0.32 |
| Residual |  |  | 3.37 |  |  |  |  |  | 1.15 |  |  |

*Note. N* = 195, 10 pictures per Study, 1,950 observations. ICC for model 2 = .67. *b* = unstandardized coefficient, *SE* = standard error.
Competence-Condition -1 = Low Competence, Competence-Condition 1 = High Competence, Meas. = Measured.
^a^Confidence intervals were computed from the profiled likelihood.

**Linear Regression Analysis Predicting a Difference Score of Voting Likelihood for Study 2a/b**

As preregistered, we also conducted a linear regression analysis. However, it needs to be considered that this was less suitable for the nested structure of our data. For the sake of completeness, we report the results here.

We firstly computed a difference score as dependent variable. Thus, we subtracted the mean voting likelihood for politicians with low perceived competence from the mean voting likelihood for politicians with high perceived competence. Supporting our hypothesis, a linear regression with objective SES as predictor and the difference score of voting likelihood as criterion resulted in a significant main effect for Study 2a/b, *b* = 0.25, *SE* = 0.08, *t*(394) = 2.95, *p* = .003, adjusted *R*^2^ = 0.02, *F*(1, 394) = 8.68, *p* = .003. This result indicates that the difference in voting likelihood between the two competence conditions was larger for voters with higher objective SES. Paralleling the results of the separate multilevel regression models, this effect was significant in Study 2a, *b* = 0.29, *SE* = 0.12, *t*(193) = 2.38, *p* = .018, adjusted *R*^2^ = 0.02, *F*(1, 193) = 5.68, *p* = .018, and failed to reach significance in Study 2b, *b* = 0.13, *SE* = 0.10, *t*(199) = 1.27, *p* = .205, adjusted *R*^2^ = 0.00, *F*(1, 199) = 1.62, *p* = .205.

In line with our assumption, across Study 2a/b the mean voting likelihood for politicians with low perceived competence (*M* = 5.20, *SD* = 1.57) was significantly lower than the mean voting likelihood for politicians with high perceived competence (*M* = 5.65, *SD* = 1.54), *t*(395) = 7.15, *p* < .001, *d* = 0.36, CI for *d* [0.26, 0.46]. This was also found in Study 2a (*M_low_* = 5.03, *SD_low_* = 1.54; *M_high_* = 5.90, *SD_high_* = 1.44, *t*(194) = 9.62, *p* < .001, *d* = 0.69, CI for *d* [0.53, 0.84]). In Study 2b, there was no significant difference in voting likelihood across the two competence-conditions (*M_low_* = 5.36, *SD_low_* = 1.59; *M_high_* = 5.40, *SD_high_* = 1.60, *t*(200) = 0.56, *p* = .577, *d* = 0.04, CI for *d* [-0.10, 0.18]).

Objective SES was no significant predictor of overall voting likelihood across Study 2a/b, *b* = 0.06, *SE* = 0.10, *t(*394) = 0.62, *p* = .536, adjusted *R*^2^ = -0.00, *F*(1, 394) = 0.38, *p* = .536.

The same results were obtained for Study 2a, *b* = -0.05, *SE* = 0.13, *t(*193) = -0.41, *p* = .679, adjusted *R*^2^ = 0.00, *F*(1, 193) = 0.17, *p* = .680, and 2b, *b* = 0.14, *SE* = 0.14, *t(*199) = 1.02, *p* = .310, adjusted *R*^2^ = 0.00, *F*(1, 199) = 1.04, *p* = .310, separately.

1. **Robustness Checks for Study 2a/b**

**Table S7**

*Parameter Estimates for Multilevel Models of Voting Likelihood for Study 2a and 2b* ***with the Adjusted Score for Objective SES*** *for Students and Apprentices*

|  | Model 1 | | | | |  | Model 2 | | | | | |  |
| --- | --- | --- | --- | --- | --- | --- | --- | --- | --- | --- | --- | --- | --- |
| Fixed effects | *b* | *SE b* | *t* | *p* | *95% CI^a^* |  | | *b* | *SE b* | *t* | *p* | *95% CI^a^* | |
| Intercept | 5.46 | 0.07 | 76.52 | <.001 | 5.32, 5.60 |  | | 5.48 | 0.07 | 75.70 | <.001 | 5.33, 5.62 | |
| Competence – Condition | 0.22 | 0.03 | 7.14 | <.001 | 0.16, 0.28 |  | |  |  |  |  |  | |
| Meas. Warmth |  |  |  |  |  |  | | 1.51 | 0.04 | 39.37 | <.001 | 1.43, 1.58 | |
| Meas. Competence |  |  |  |  |  |  | | 1.33 | 0.04 | 30.81 | <.001 | 1.24, 1.41 | |
| Adj. SES | 0.06 | 0.08 | 0.74 | .462 | -0.09, 0.20 |  | | 0.06 | 0.08 | 0.85 | .398 | -0.08, 0.21 | |
| Source | -0.11 | 0.14 | -0.79 | .432 | -0.40, 0.17 |  | | -0.14 | 0.14 | -0.94 | .347 | -0.42, 0.15 | |
| Meas. Warmth x Source |  |  |  |  |  |  | | 0.18 | 0.08 | 2.31 | .021 | 0.03, 0.33 | |
| Meas. Competence  x Source |  |  |  |  |  |  | | -0.11 | 0.09 | -1.23 | .220 | -0.27, 0.06 | |
| Meas. Warmth x  Meas. Competence |  |  |  |  |  |  | | 0.26 | 0.04 | 6.98 | <.001 | 0.19, 0.34 | |
| Competence – Condition x  Adj. SES | 0.10 | 0.03 | 3.11 | .002 | 0.04, 0.16 |  | |  |  |  |  |  | |
| Meas. Warmth x Adj. SES |  |  |  |  |  |  | | -0.03 | 0.04 | -0.85 | .395 | -0.11, 0.04 | |

**Table S7 (continued)**

| Meas. Competence x  Adj. SES |  |  |  |  |  |  | 0.16 | 0.04 | 3.56 | <.001 | 0.07, 0.25 |
| --- | --- | --- | --- | --- | --- | --- | --- | --- | --- | --- | --- |
| Adj. SES x Source |  |  |  |  |  |  | 0.13 | 0.15 | 0.85 | .394 | -0.17, 0.43 |
| Meas. Warmth x Adj. SES  x Source |  |  |  |  |  |  | -0.06 | 0.08 | -0.72 | .472 | -0.22, 0.10 |
| Meas. Competence x  Adj. SES x Source |  |  |  |  |  |  | 0.11 | 0.09 | 1.18 | .238 | -0.07, 0.28 |

| Random effects | |  |  |  |  |  |  |  |  |  |  |
| --- | --- | --- | --- | --- | --- | --- | --- | --- | --- | --- | --- |
| Intercept |  |  | 1.65 |  |  |  |  |  | 1.90 |  |  |
| Competence-Condition |  |  | 0.00 | 0.06 |  |  |  |  |  |  |  |
| Meas. Competence |  |  |  |  |  |  |  |  | 0.29 | 0.26 |  |
| Meas. Warmth |  |  |  |  |  |  |  |  | 0.28 | 0.04 | 0.01 |
| Residual |  |  | 3.74 |  |  |  |  |  | 1.21 |  |  |

*Note. N* = 396, 10 pictures per Study, 3,960 observations. *B* = unstandardized coefficient, *SE* = standard error.
Competence-Condition -1 = Low Competence, Competence-Condition 1 = High Competence, Meas. = Measured, Adj. SES = adjusted score of objective socio-economic status. Source 0 = Study 2a, Source 1 = Study 2b.
^a^Confidence intervals were computed from the profiled likelihood.

**Table S8**

*Parameter Estimates for Multilevel Models of Voting Likelihood for Study 2a and 2b* ***with Educational Attainment as Single Indicator*** *of Objective SES*

|  | Model 1 | | | | |  | Model 2 | | | | | |  |
| --- | --- | --- | --- | --- | --- | --- | --- | --- | --- | --- | --- | --- | --- |
| Fixed effects | *b* | *SE b* | *t* | *p* | *95% CI^a^* |  | | *b* | *SE b* | *t* | *p* | *95% CI^a^* | |
| Intercept | 5.46 | 0.07 | 76.59 | <.001 | 5.32, 5.60 |  | | 5.47 | 0.07 | 75.67 | <.001 | 5.33, 5.61 | |
| Competence – Condition | 0.22 | 0.03 | 7.05 | <.001 | 0.16, 0.28 |  | |  |  |  |  |  | |
| Meas. Warmth |  |  |  |  |  |  | | 1.51 | 0.04 | 39.59 | <.001 | 1.44, 1.59 | |
| Meas. Competence |  |  |  |  |  |  | | 1.32 | 0.04 | 30.45 | <.001 | 1.24, 1.41 | |
| Education | -0.06 | 0.06 | -0.92 | .360 | -0.18, 0.07 |  | | -0.05 | 0.06 | -0.78 | .436 | -0.18, 0.08 | |
| Source | -0.10 | 0.14 | -0.71 | .476 | -0.40, 0.20 |  | | -0.14 | 0.14 | -0.96 | .338 | -0.42, 0.15 | |
| Meas. Warmth x Source |  |  |  |  |  |  | | 0.18 | 0.08 | 2.40 | .017 | 0.03, 0.33 | |
| Meas. Competence  x Source |  |  |  |  |  |  | | -0.18 | 0.09 | -2.11 | .036 | -0.35, -0.01 | |
| Meas. Warmth x  Meas. Competence |  |  |  |  |  |  | | 0.26 | 0.04 | 6.98 | <.001 | 0.19, 0.34 | |
| Competence – Condition x  Education | 0.02 | 0.03 | 0.72 | .472 | -0.03, 0.07 |  | |  |  |  |  |  | |
| Meas. Warmth x Education |  |  |  |  |  |  | | 0.01 | 0.03 | 0.22 | .827 | -0.06, 0.08 | |

**Table S8 (continued)**

| Meas. Competence x  Education |  |  |  |  |  |  | 0.09 | 0.04 | 2.18 | .030 | 0.01, 0.17 |
| --- | --- | --- | --- | --- | --- | --- | --- | --- | --- | --- | --- |
| Education x Source |  |  |  |  |  |  | -0.03 | 0.13 | -0.21 | .837 | -0.28, 0.22 |
| Meas. Warmth x Education  x Source |  |  |  |  |  |  | -0.01 | 0.07 | -0.15 | .885 | -0.15, 0.13 |
| Meas. Competence x  Education x Source |  |  |  |  |  |  | -0.10 | 0.08 | -1.19 | .236 | -0.25, 0.06 |

| Random effects | |  |  |  |  |  |  |  |  |  |  |
| --- | --- | --- | --- | --- | --- | --- | --- | --- | --- | --- | --- |
| Intercept |  |  | 1.64 |  |  |  |  |  | 1.90 |  |  |
| Competence-Condition |  |  | 0.01 | 0.05 |  |  |  |  |  |  |  |
| Meas. Competence |  |  |  |  |  |  |  |  | 0.31 | 0.28 |  |
| Meas. Warmth |  |  |  |  |  |  |  |  | 0.28 | 0.04 | -0.01 |
| Residual |  |  | 3.74 |  |  |  |  |  | 1.21 |  |  |

*Note. N* = 396, 10 pictures per Study, 3,960 observations. ICC for model 1(2) = .31 (.64). *b* = unstandardized coefficient, *SE* = standard error.
Competence-Condition -1 = Low Competence, Competence-Condition 1 = High Competence, Meas. = Measured. Source 0 = Study 2a, Source 1 = Study 2b.
^a^Confidence intervals were computed from the profiled likelihood.

**Table S9**

*Parameter Estimates for Multilevel Models of Voting Likelihood for Study 2a and 2b with* ***Household Income as Single Indicator*** *of Objective*

*SES*

|  | Model 1 | | | | |  | Model 2 | | | | | |
| --- | --- | --- | --- | --- | --- | --- | --- | --- | --- | --- | --- | --- |
| Fixed effects | *b* | *SE b* | *t* | *p* | *95% CI^a^* |  | | *b* | *SE b* | *t* | *p* | *95% CI^a^* |
| Intercept | 5.46 | 0.07 | 76.71 | <.001 | 5.32, 5.60 |  | | 5.50 | 0.07 | 74.50 | <.001 | 5.35, 5.64 |
| Competence – Condition | 0.22 | 0.03 | 7.18 | <.001 | 0.16, 0.28 |  | |  |  |  |  |  |
| Meas. Warmth |  |  |  |  |  |  | | 1.49 | 0.04 | 38.16 | <.001 | 1.42, 1.57 |
| Meas. Competence |  |  |  |  |  |  | | 1.35 | 0.04 | 30.52 | <.001 | 1.26, 1.43 |
| Income | 0.07 | 0.04 | 1.76 | .080 | -0.01, 0.15 |  | | 0.07 | 0.04 | 1.72 | .086 | -0.01, 0.15 |
| Source | -0.06 | 0.15 | -0.43 | .667 | -0.37, 0.24 |  | | -0.09 | 0.15 | -0.60 | .552 | -0.38, 0.20 |
| Meas. Warmth x Source |  |  |  |  |  |  | | 0.16 | 0.08 | 2.08 | .038 | 0.01, 0.32 |
| Meas. Competence  x Source |  |  |  |  |  |  | | -0.10 | 0.09 | -1.08 | .281 | -0.27, 0.08 |
| Meas. Warmth x  Meas. Competence |  |  |  |  |  |  | | 0.26 | 0.04 | 7.00 | <.001 | 0.19, 0.34 |
| Competence – Condition x  Income | 0.06 | 0.02 | 3.64 | <.001 | 0.03, 0.09 |  | |  |  |  |  |  |
| Meas. Warmth x Income |  |  |  |  |  |  | | -0.03 | 0.02 | -1.30 | .193 | -0.07, 0.01 |
| Meas. Competence x  Income |  |  |  |  |  |  | | 0.07 | 0.02 | 2.82 | .005 | 0.02, 0.11 |
| Income x Source |  |  |  |  |  |  | | 0.13 | 0.08 | 1.60 | .109 | -0.03, 0.28 |

**Table S9 (continued)**

| Meas. Warmth x Income  x Source |  |  |  |  |  |  | -0.06 | 0.04 | -1.36 | .174 | -0.14, 0.03 |
| --- | --- | --- | --- | --- | --- | --- | --- | --- | --- | --- | --- |
| Meas. Competence x  Income x Source |  |  |  |  |  |  | 0.10 | 0.05 | 2.13 | .034 | 0.01, 0.19 |

| Random effects | |  |  |  |  |  |  |  |  |  |  |
| --- | --- | --- | --- | --- | --- | --- | --- | --- | --- | --- | --- |
| Intercept |  |  | 1.63 |  |  |  |  |  | 1.87 |  |  |
| Competence-Condition |  |  | 0.00 | -0.21 |  |  |  |  |  |  |  |
| Meas. Competence |  |  |  |  |  |  |  |  | 0.29 | 0.25 |  |
| Meas. Warmth |  |  |  |  |  |  |  |  | 0.28 | 0.05 | 0.04 |
| Residual |  |  | 3.74 |  |  |  |  |  | 1.22 |  |  |

*Note. N* = 396, 10 pictures per Study, 3,960 observations. ICC for model 1(2) = .30(.64). *b* = unstandardized coefficient, *SE* = standard error.
Competence-Condition -1 = Low Competence, Competence-Condition 1 = High Competence, Meas. = Measured. Income = Household Income. Source 0 = Study 2a, Source 1 = Study 2b.
^a^Confidence intervals were computed from the profiled likelihood

**Table S10**

*Parameter Estimates for Multilevel Models of Voting Likelihood for Study 2a and 2b with* ***Subjective Social Status as Indicator*** *of Social Class*

|  | Model 1 | | | | |  | Model 2 | | | | | |
| --- | --- | --- | --- | --- | --- | --- | --- | --- | --- | --- | --- | --- |
| Fixed effects | *b* | *SE b* | *t* | *p* | *95% CI^a^* |  | | *b* | *SE b* | *t* | *p* | *95% CI^a^* |
| Intercept | 5.46 | 0.07 | 76.66 | <.001 | 5.32, 5.60 |  | | 5.48 | 0.07 | 76.78 | <.001 | 5.33, 5.62 |
| Competence – Condition | 0.22 | 0.03 | 7.06 | <.001 | 0.16, 0.28 |  | |  |  |  |  |  |
| Meas. Warmth |  |  |  |  |  |  | | 1.50 | 0.04 | 39.60 | <.001 | 1.43, 1.58 |
| Meas. Competence |  |  |  |  |  |  | | 1.33 | 0.04 | 30.91 | <.001 | 1.24, 1.41 |
| SSS | 0.06 | 0.05 | 1.37 | .172 | -0.03, 0.16 |  | | 0.06 | 0.05 | 1.32 | .187 | -0.03, 0.15 |
| Source | -0.11 | 0.14 | -0.80 | .424 | -0.41, 0.18 |  | | -0.14 | 0.14 | -0.99 | .323 | -0.42, 0.14 |
| Meas. Warmth x Source |  |  |  |  |  |  | | 0.20 | 0.08 | 2.69 | .008 | 0.05, 0.35 |
| Meas. Competence  x Source |  |  |  |  |  |  | | -0.17 | 0.09 | -1.94 | .053 | -0.34, 0.00 |
| Meas. Warmth x  Meas. Competence |  |  |  |  |  |  | | 0.27 | 0.04 | 7.04 | <.001 | 0.19, 0.34 |
| Competence – Condition x  SSS | 0.03 | 0.02 | 1.30 | .196 | -0.01, 0.07 |  | |  |  |  |  |  |
| Meas. Warmth x SSS |  |  |  |  |  |  | | 0.03 | 0.02 | 1.21 | .223 | -0.02, 0.08 |

**Table S10 (continued)**

| Meas. Competence x  SSS |  |  |  |  |  |  | 0.01 | 0.03 | 0.19 | .851 | -0.05, 0.06 |
| --- | --- | --- | --- | --- | --- | --- | --- | --- | --- | --- | --- |
| SSS x Source |  |  |  |  |  |  | 0.15 | 0.09 | 1.60 | .110 | -0.03, 0.34 |
| Meas. Warmth x SSS  x Source |  |  |  |  |  |  | -0.08 | 0.05 | -1.63 | .105 | -0.18, 0.02 |
| Meas. Competence x  SSS x Source |  |  |  |  |  |  | 0.10 | 0.06 | 1.77 | .078 | -0.01, 0.21 |

| Random effects | |  |  |  |  |  |  |  |  |  |  |
| --- | --- | --- | --- | --- | --- | --- | --- | --- | --- | --- | --- |
| Intercept |  |  | 1.64 |  |  |  |  |  | 1.88 |  |  |
| Competence-Condition |  |  | 0.01 | 0.01 |  |  |  |  |  |  |  |
| Meas. Competence |  |  |  |  |  |  |  |  | 0.30 | 0.26 |  |
| Meas. Warmth |  |  |  |  |  |  |  |  | 0.28 | 0.04 | 0.02 |
| Residual |  |  | 3.74 |  |  |  |  |  | 1.22 |  |  |

*Note. N* = 396, 10 pictures per Study, 3,960 observations. ICC for model 1(2) = .31(.64). *b* = unstandardized coefficient, *SE* = standard error.
Competence-Condition -1 = Low Competence, Competence-Condition 1 = High Competence, Meas. = Measured, SSS = Subjective Social Status. Source 0 = Study 2a, Source 1 = Study 2b.
^a^Confidence intervals were computed from the profiled likelihood.

.

**Table S11**

*Parameter Estimates for Multilevel Model of Voting Likelihood for Study 2a and 2b* ***with Crossed Random Effects***

| Fixed effects | *b* | *SE b* | *t* | *p* | *95% CI^a^* |
| --- | --- | --- | --- | --- | --- |
| Intercept | 5.47 | 0.08 | 65.23 | <.001 | 5.31, 5.64 |
| Meas. Warmth | 1.56 | 0.05 | 31.87 | <.001 | 1.46, 1.66 |
| Meas. Competence | 1.18 | 0.05 | 23.99 | <.001 | 1.08, 1.29 |
| Obj. SES | 0.05 | 0.10 | 0.53 | .598 | -0.14, 0.17 |
| Source | -0.13 | 0.17 | -0.79 | .431 | -0.47, 0.20 |
| Meas. Warmth x Source | 0.24 | 0.10 | 2.46 | .019 | 0.04, 0.44 |
| Meas. Competence  x Source | -0.17 | 0.10 | -1.72 | .093 | -0.37, 0.03 |
| Meas. Warmth x  Meas. Competence | 0.22 | 0.04 | 5.43 | <.001 | 0.14, 0.31 |
| Meas. Warmth x Obj. SES | -0.04 | 0.05 | -0.70 | .485 | -0.14, 0.07 |
| Meas. Competence x  Obj. SES | 0.18 | 0.06 | 3.10 | .002 | 0.07, 0.30 |
| Obj. SES x Source | 0.16 | 0.19 | 0.81 | .418 | -0.22, 0.54 |
| Meas. Warmth x Obj. SES  x Source | -0.09 | 0.10 | -0.88 | .378 | -0.30, 0.11 |
| Meas. Competence x  Obj. SES x Source | 0.07 | 0.12 | 0.59 | .553 | -0.16, 0.30 |
| Random effects |  |  |  |  |  |
| Participant |  |  |  |  |  |
| Intercept | 1.90 |  |  |  |  |
| Meas. Competence | 0.30 | 0.25 |  |  |  |
| Meas. Warmth | 0.27 | 0.05 | -0.04 |  |  |
| Political Candidate |  |  |  |  |  |
| Intercept | 0.04 |  |  |  |  |
| Meas. Competence | 0.01 | -0.45 |  |  |  |

**Table S11 (continued)**

| Meas. Warmth | 0.02 | 0.20 | -0.85 |  |  |
| --- | --- | --- | --- | --- | --- |
| Residual | 1.18 |  |  |  |  |

*Note. N* = 396, 10 pictures per Study, 3,960 observations. ICC = .65. *b* = unstandardized coefficient, *SE* = standard error. Meas. = Measured. Source 0 = Study 2a, Source 1 = Study 2b.
^a^Confidence intervals were computed from the profiled likelihood.

1. **Exploratory Analyses for Study 2a/b and 3: Status Influences on Competence Perception**

**Study 2a/b.** To address the alternative account that SES does not influence the weighting of competence but that social classes differ in their perception of competence, as one might expect for competence schematics and aschematics (Green & Sedikides, 2001), we conducted a multilevel moderated mediation analysis. We included the effect of pretest competence ratings on measured competence in Studies 2a/b as well as the effects of both on voting likelihood (see Figure S1). Participants’ SES was included as moderator of all three effects. Measured warmth was included as covariate. All models had random intercepts on the participant level and all variables were standardized (grand-mean). As depicted in figure 2, SES moderated the relationship between the competence measures in the pretest and main study. Simple slope analyses indicated that while generally politicians who looked competent according to the pretest were also rated higher in competence in the main study than politicians who looked less competent, this effect was more pronounced for participants of high SES (+ 1 SD: *b* = 0.28, *SE* = 0.02, *z* = 15.60, *p* < .001, 95% CI [0.24, 0.31]) rather than low SES (-1 *SD*: *b* = 0.41, *SE* = 0.02, *z* = 17.64, *p* < .001, 95% CI[0.36, 0.45]). This might suggest that, as expected for schematics vs. aschematics (e.g., Shoda & McConnell, 2013),

participants of high SES differentiated politicians more along the competence dimension than participants of low SES. Importantly, however, this does not explain the higher weighting of competence for voting likelihood among participants high in SES, as the hypothesized interaction was still significant. Moreover, the indirect effect of pretest competence via measured competence including both moderating effects of SES significantly predicted voting likelihood, *b* = 0.19, *SE* = 0.02, z = 12.65, *p* < .001, 95% CI [0.16, 0.22]. Complete results are presented in Table S12.

**Figure S1. Unstandardized Regression Coefficients from the Multilevel Moderated Mediation Analysis of Studies 2a/b*.***

Measured Warmth

Measured Competence

0.59***

0.40***

0.34***

0.00

0.07***

0.07***

-0.02

SES

-0.02

0.01

0.06***

Voting Likelihood

Pretest Competence

*Note.* All variables were standardized at the grand mean. *** *p* < .001, ** *p* < .01, * *p* < .05

**Status Influences on Competence Perception for Study 2a and 2b:
A Multilevel Moderated Mediation Analysis**

**Table S12**

|  | Full Mediation Model | | | | |
| --- | --- | --- | --- | --- | --- |
| Levels  and Components | *b* | *SE b* | *z* | *p* | *95% CI* |
| Level 1 |  |  |  |  |  |
| Regressions |  |  |  |  |  |
| Comp. ~ |  |  |  |  |  |
| Pre. Comp. (a1) | 0.34 | 0.02 | 23.08 | < .001 | 0.31, 0.37 |
| Pre. Comp. x   Obj. SES (a3) | 0.07 | 0.01 | 4.62 | < .001 | 0.04, 0.09 |
| Voting ~ |  |  |  |  |  |
| Pre. Comp. (c1) | 0.06 | 0.01 | 6.44 | < .001 | 0.04, 0.08 |
| Pre. Comp. x   Obj. SES (c3) | -0.02 | 0.01 | -1.86 | .063 | -0.03, 0.00 |
| Comp. (b1) | 0.40 | 0.02 | 22.49 | < .001 | 0.36, 0.43 |
| Comp. x  Obj. SES (b2) | 0.07 | 0.02 | 4.85 | < .001 | 0.04, 0.10 |
| Warmth | 0.59 | 0.02 | 36.35 | < .001 | 0.56, 0.62 |
| Warmth x  Obj. SES | -0.02 | 0.02 | -1.05 | .296 | -0.05, 0.01 |
| Intercepts |  |  |  |  |  |
| Comp. | 0.00 |  |  |  | 0.00, 0.00 |
| Voting | 0.00 |  |  |  | 0.00, 0.00 |
| Comp. x  Obj. SES | 0.00 | 0.03 | 0.06 | .954 | -0.06, 0.06 |
| Variances |  |  |  |  |  |
| Comp. | 0.67 | 0.03 | 26.35 | < .001 | 0.62, 0.72 |
| Voting | 0.28 | 0.01 | 20.87 | < .001 | 0.26, 0.31 |
| Comp. x  Obj. SES | 1.03 | 0.13 | 7.85 | < .001 | 0.77, 1.28 |

*Full Results of the Mediation Model for Study 2a and 2b*

**Table S12 (continued)**

| Level 2 |  |  |  |  |  |
| --- | --- | --- | --- | --- | --- |
| Regressions |  |  |  |  |  |
| Comp. ~ |  |  |  |  |  |
| Obj. SES (a2) | 0.00 | 0.03 | 0.11 | .909 | -0.05, 0.06 |
| Voting ~ |  |  |  |  |  |
| Obj. SES (c2) | 0.01 | 0.03 | 0.45 | .654 | -0.05, 0.07 |
| Covariances |  |  |  |  |  |
| Comp. ~ |  |  |  |  |  |
| Voting | 0.12 | 0.02 | 5.46 | < .001 | 0.08, 0.17 |
| Intercepts |  |  |  |  |  |
| Comp. | 0.00 | 0.03 | 0.00 | .999 | -0.05, 0.05 |
| Voting | 0.00 | 0.02 | 0.00 | .997 | -0.06, 0.06 |
| Warmth | -0.00 | 0.00 | -0.00 | 1.00 | -0.01, 0.01 |
| Variances |  |  |  |  |  |
| Comp. | 0.21 | 0.03 | 7.07 | < .001 | 0.15, 0.27 |
| Voting | 0.24 | 0.02 | 9.89 | < .001 | 0.19, 0.29 |
| Warmth | 0.14 | 0.00 | 39.71 | < .001 | 0.13, 0.14 |
| Defined Parameters |  |  |  |  |  |
| Indirect  (a1 + a3) *  (b1 + b2) | 0.19 | 0.02 | 12.65 | < .001 | 0.16, 0.22 |
| Direct  (c1 + c3) | 0.05 | 0.01 | 3.78 | < .001 | 0.02, 0.07 |
| Total | 0.24 | 0.02 | 15.55 | < .001 | 0.21, 0.27 |
| Simple Slopes |  |  |  |  |  |
| Obj. SES -1SD | 0.28 | 0.02 | 15.60 | < .001 | 0.24, 0.31 |
| Obj. SES Mean | 0.34 | 0.02 | 23.08 | < .001 | 0.31, 0.37 |
| Obj. SES -1SD | 0.41 | 0.02 | 17.64 | < .001 | 0.36, 0.45 |

*Note. N* (level 2) = 10 pictures per Study*, N* (level 1) = 396, observations used = 3,960. *B* = unstandardized coefficient, SE = standard error. Pre. Comp = Competence ratings from pretest, Comp = Perceived competence as measured in Study 2a and b. All variables were z-standardized. Standard errors were computed with the Huber-White method.

**Study 3.** Parallel to Studies 2a/b, we conducted a multilevel moderated mediation analysis for Study 3. Again, SES moderated the effect of competence ratings from the independent study on measured competence indicating that as in Studies 2a/b participants of higher SES differentiated others to a larger extent with regard to competence than participants with lower SES (see Figure S2). Still the hypothesized interaction between SES and measured competence on voting likelihood was significant.

Moreover, the indirect effect of competence ratings from the independent study via measured competence including both moderating effects of SES significantly predicted voting likelihood, *b* = .10, *SE* = .01, *z* = 12.83, *p* < .001, CI [0.08, 0.12] (see Table S13 for complete results).

**Figure S2. Unstandardized Regression Coefficients from the Multilevel Moderated Mediation Analysis of Study 3*.***

Measured Warmth

Measured Competence

0.28***

0.27***

0.31***

-0.02

0.02*

0.02**

-0.01

SES

0.04

0.00

0.11***

Voting Likelihood

New Competence

*Note.* All variables were standardized at the grand mean. New competence refers to ratings of competence from the independent study.

*** *p* < .001, ** *p* < .01, * *p* < .05

**Status Influences on Competence Perception for Study 3:
A Multilevel Moderated Mediation Analysis**

**Table S13**

|  | Full Mediation Model | | | | |
| --- | --- | --- | --- | --- | --- |
| Levels  and Components | *b* | *SE b* | *z* | *p* | *95% CI* |
| Level 1 |  |  |  |  |  |
| Regressions |  |  |  |  |  |
| Comp. ~ |  |  |  |  |  |
| New Comp. (a1) | 0.31 | 0.01 | 32.04 | < .001 | 0.29, 0.33 |
| New Comp. x   Obj. SES (a3) | 0.02 | 0.01 | 2.17 | .030 | 0.00, 0.04 |
| Voting ~ |  |  |  |  |  |
| New Comp. (c1) | 0.11 | 0.01 | 14.10 | < .001 | 0.10, 0.13 |
| New Comp. x   Obj. SES (c3) | 0.00 | 0.01 | 0.39 | .699 | -0.01, 0.02 |
| Comp. (b1) | 0.27 | 0.01 | 20.52 | < .001 | 0.25, 0.30 |
| Comp. x  Obj. SES (b2) | 0.02 | 0.01 | 2.14 | .032 | 0.00, 0.05 |
| Warmth | 0.28 | 0.01 | 21.80 | < .001 | 0.26, 0.31 |
| Warmth x  Obj. SES | -0.01 | 0.01 | -0.85 | .394 | -0.03, 0.01 |
| Intercepts |  |  |  |  |  |
| Comp. | 0.00 |  |  |  | 0.00, 0.00 |
| Voting | 0.00 |  |  |  | 0.00, 0.00 |
| Comp. x  Obj. SES | -0.02 | 0.03 | -0.70 | .484 | -0.07, 0.03 |
| Variances |  |  |  |  |  |
| Comp. | 0.62 | 0.02 | 33.07 | < .001 | 0.58, 0.66 |
| Voting | 0.52 | 0.02 | 29.42 | < .001 | 0.48, 0.55 |
| Comp. x  Obj. SES | 0.89 | 0.07 | 12.42 | < .001 | 0.75, 1.04 |

*Full Results of the Mediation Model for Study 3 Including Competence Ratings from Independent Sample*

**Table S13 (continued)**

| Level 2 |  |  |  |  |  |
| --- | --- | --- | --- | --- | --- |
| Regressions |  |  |  |  |  |
| Comp. ~ |  |  |  |  |  |
| Obj. SES (a2) | -0.02 | 0.03 | -0.70 | .485 | -0.07, 0.03 |
| Voting ~ |  |  |  |  |  |
| Obj. SES (c2) | 0.04 | 0.03 | 1.43 | .153 | -0.01, 0.09 |
| Covariances |  |  |  |  |  |
| Comp. ~ |  |  |  |  |  |
| Voting | 0.15 | 0.02 | 6.97 | < .001 | 0.10, 0.19 |
| Intercepts |  |  |  |  |  |
| Comp. | -0.00 | 0.03 | 0.00 | 1.00 | -0.05, 0.05 |
| Voting | 0.00 | 0.03 | 0.02 | .988 | -0.05, 0.05 |
| Warmth | 0.00 | 0.00 | 0.00 | 1.00 | -0.00, 0.00 |
| Variances |  |  |  |  |  |
| Comp. | 0.28 | 0.03 | 9.98 | < .001 | 0.23, 0.34 |
| Voting | 0.28 | 0.02 | 12.29 | < .001 | 0.24, 0.33 |
| Warmth | 0.24 | 0.00 | 403.59 | < .001 | 0.24, 0.24 |
| Defined Parameters |  |  |  |  |  |
| Indirect  (a1 + a3) *  (b1 + b2) | 0.10 | 0.01 | 12.83 | < .001 | 0.08, 0.12 |
| Direct  (c1 + c3) | 0.12 | 0.01 | 10.26 | < .001 | 0.09, 0.14 |
| Total | 0.22 | 0.01 | 17.52 | < .001 | 0.19, 0.24 |
| Simple Slopes |  |  |  |  |  |
| Obj. SES -1SD | 0.29 | 0.01 | 23.11 | < .001 | 0.27, 0.32 |
| Obj. SES Mean | 0.31 | 0.01 | 32.04 | < .001 | 0.29, 0.33 |
| Obj. SES -1SD | 0.33 | 0.01 | 32.21 | < .001 | 0.31, 0.36 |

*Note. N* = 400, 32 pictures*,* 12,800 observations. *B* = unstandardized coefficient, SE = standard error. New Comp = Competence ratings from independent sample (*n* = 30). Comp = Perceived competence as measured in Study 3. All variables were z-standardized. Standard errors were computed with the Huber-White method.

1. **Robustness Checks for Study 3**

**Table S14**

*Parameter Estimates for Multilevel Models of Voting Likelihood with* ***Crossed Random Effects*** *of Participants and Political Candidates*

*(Study 3)*

|  | Model 1 | | | | |  | Model 2 | | | | | |
| --- | --- | --- | --- | --- | --- | --- | --- | --- | --- | --- | --- | --- |
| Fixed effects | *b* | *SE b* | *t* | *p* | *95% CI* |  | | *b* | *SE b* | *t* | *p* | *95% CI* |
| Intercept | 5.11 | 0.10 | 51.82 | <.001 | 4.92, 5.31 |  | | 5.12 | 0.09 | 60.16 | <.001 | 4.95, 5.29 |
| Warmth |  |  |  |  |  |  | | 0.69 | 0.03 | 19.85 | <.001 | 0.62, 0.75 |
| Competence | 0.97 | 0.04 | 21.74 | <.001 | 0.88, 1.06 |  | | 0.71 | 0.04 | 19.32 | <.001 | 0.64, 0.79 |
| Obj. SES | 0.12 | 0.08 | 1.42 | .156 | -0.05, 0.29 |  | | 0.12 | 0.08 | 1.42 | .157 | -0.05, 0.28 |
| Warmth x Obj. SES |  |  |  |  |  |  | | -0.02 | 0.04 | -0.61 | .543 | -0.10, 0.05 |
| Competence x Obj. SES | 0.04 | 0.04 | 0.92 | .356 | -0.04, 0.11 |  | | 0.07 | 0.04 | 1.90 | .059 | -0.00, 0.15 |

| Random effects | |  |  |  |  |  |  |  |  |  |  |
| --- | --- | --- | --- | --- | --- | --- | --- | --- | --- | --- | --- |
| Participant |  |  |  |  |  |  |  |  |  |  |  |
| Intercept |  |  | 1.59 |  |  |  |  |  | 1.60 |  |  |
| Warmth |  |  |  |  |  |  |  |  | 0.15 | 0.05 |  |
| Competence |  |  | 0.16 | 0.28 |  |  |  |  | 0.13 | 0.31 | -0.32 |

**Table S14 (continued)**

| Political Candidate |  |  |  |  |  |  |  |  |  |  |  |
| --- | --- | --- | --- | --- | --- | --- | --- | --- | --- | --- | --- |
| Intercept |  |  | 0.18 |  |  |  |  |  | 0.10 |  |  |
| Warmth |  |  |  |  |  |  |  |  | 0.01 | 0.15 |  |
| Competence |  |  | 0.03 | 0.12 |  |  |  |  | 0.01 | -0.12 | 0.14 |
| Residual |  |  | 2.86 |  |  |  |  |  | 2.58 |  |  |

*Note. N* = 400, 32 pictures*,* 12,800 observations. Warmth and competence refer to the perceived traits as measured in Study 3. *B* = unstandardized coefficient, *SE* = standard error

**Table S15**

*Parameter Estimates for Multilevel Models of Voting Likelihood* ***Controlling for the Order of the Assessment of Warmth and Competence***

*(Study 3)*

|  | Model 1 | | | | |  | Model 2 | | | | | |
| --- | --- | --- | --- | --- | --- | --- | --- | --- | --- | --- | --- | --- |
| Fixed effects | *b* | *SE b* | *t* | *P* | *95% CI^a^* |  | | *b* | *SE b* | *t* | *p* | *95% CI^a^* |
| Intercept | 5.08 | 0.90 | 56.40 | <.001 | 4.90, 5.26 |  | | 5.08 | 0.09 | 56.53 | <.001 | 4.90, 5.25 |
| Warmth |  |  |  |  |  |  | | 0.72 | 0.03 | 25.31 | <.001 | 0.67, 0.78 |
| Competence | 1.10 | 0.03 | 36.60 | <.001 | 1.04, 1.16 |  | | 0.83 | 0.03 | 28.42 | <.001 | 0.77, 0.88 |
| Order | 0.08 | 0.13 | 0.61 | .540 | -0.17, 0.33 |  | | 0.08 | 0.13 | 0.67 | .507 | -0.17, 0.34 |
| Obj. SES | 0.11 | 0.08 | 1.35 | .179 | -0.05, 0.28 |  | | 0.11 | 0.08 | 1.34 | .181 | -0.05, 0.28 |
| Warmth x Obj. SES |  |  |  |  |  |  | | -0.02 | 0.04 | -0.41 | .681 | -0.09, 0.06 |
| Competence x Obj. SES | 0.03 | 0.04 | 0.86 | .392 | -0.04, 0.11 |  | | 0.08 | 0.04 | 1.95 | .052 | -0.00, 0.15 |

| Random effects | |  |  |  |  |  |  |  |  |  |  |
| --- | --- | --- | --- | --- | --- | --- | --- | --- | --- | --- | --- |
| Intercept |  |  | 1.58 |  |  |  |  |  | 1.59 |  |  |
| Warmth |  |  |  |  |  |  |  |  | 0.14 | 0.05 |  |
| Competence |  |  | 0.16 | 0.29 |  |  |  |  | 0.14 | 0.31 | -0.33 |

**Table S15 (continued)**

| Residual |  |  | 3.06 |  |  |  |  |  | 2.69 |  |  |
| --- | --- | --- | --- | --- | --- | --- | --- | --- | --- | --- | --- |

*Note. N* = 400, 32 pictures*,* 12,800 observations. Warmth and competence refer to the perceived traits as measured in Study 3. Order 1 = first competence, then warmth, Order 2 = first warmth, then competence. *B* = unstandardized coefficient, *SE* = standard error
^a^Confidence intervals were computed from the profiled likelihood.

***Extreme cases.*** We investigated if the results of Studies 2a/b which used pictures from the lower and higher ends of the competence spectrum could be replicated. Looking only at the five politicians with the lowest and the highest perceived competence^[[1]](#footnote-1)^ we found a significant interaction between competence and SES when controlling for warmth and its interaction with SES, *b* = 0.12, *SE* = 0.05, *t* = 2.26, *p* < .024, 95% CI [0.02, 0.23]. Looking only at politicians at the extreme ends, as were used in Studies 2a/b, we replicate the effect of Studies 2a/b even when competence is not made salient.

**(7) Independent Ratings of Dominance, Competence, Warmth as Predictors of Voting Likelihood in Study 3**

**Table S16**

*Parameter Estimates for ML Models of Voting Likelihood with Dominance, Competence, and Warmth Ratings from Independent Study*

|  | Model 1 | | | | |  | Model 2 | | | | | |
| --- | --- | --- | --- | --- | --- | --- | --- | --- | --- | --- | --- | --- |
| Fixed effects | *b* | *SE b* | *t* | *p* | *95% CI* |  | | *b* | *SE b* | *t* | *p* | *95% CI* |
| Intercept | 5.12 | 0.06 | 79.22 | <.001 | 4.99, 5.25 |  | | 5.11 | 0.06 | 79.22 | <.001 | 4.99, 5.25 |
| Warmth |  |  |  |  |  |  | | 0.49 | 0.40 | 12.69 | <.001 | 0.41, 0.57 |
| Dominance |  |  |  |  |  |  | | -0.02 | 0.04 | -0.50 | .619 | -0.10, 0.06 |
| Competence | 1.65 | 0.05 | 33.26 | <.001 | 1.55, 1.74 |  | | 1.53 | 0.05 | 30.85 | <.001 | 1.44, 1.63 |
| Obj. SES | 0.12 | 0.08 | 1.40 | .162 | -0.05, 0.28 |  | | 0.12 | 0.08 | 1.40 | .162 | -0.05, 0.28 |
| Warmth x Obj. SES |  |  |  |  |  |  | | -0.01 | 0.05 | -0.24 | .810 | -0.11, 0.09 |
| Dominance x Obj. SES |  |  |  |  |  |  | | 0.13 | 0.05 | 2.50 | .013 | 0.03, 0.23 |
| Competence x Obj. SES | 0.15 | 0.06 | 2.36 | .018 | 0.03, 0.28 |  | | 0.18 | 0.06 | 2.78 | .005 | 0.05, 0.31 |

| **Table S16 (continued)**  Random effects | |  |  |  |  |  |  |  |  |  |  |
| --- | --- | --- | --- | --- | --- | --- | --- | --- | --- | --- | --- |
| Intercept |  |  | 1.56 |  |  |  |  |  | 1.56 |  |  |
| Residual |  |  | 3.54 |  |  |  |  |  | 3.48 |  |  |

*Note. N* = 400, 32 pictures*,* 12,800 observations. Warmth, dominance and competence refer to the ratings of an independent sample (*N* = 96); SES and voting likelihood are from Study 3. *B* = unstandardized coefficient, *SE* = standard error.

**Simple Slopes Analyses fort the Interaction of Dominance and SES.** SES significantly moderated the effect of perceived dominance, *b* = 0.13, *SE* = 0.05, *t* = 2.50, *p* = .013, 95% CI [0.03, 0.23]). Higher perceived dominance was significantly associated with *lower* voting likelihood at low levels of SES (-1 SD, *b* = -0.12, *SE* = 0.06, *t* = -2.12, *p* = .034, 95% CI [-0.23, -0.01]). The relationship between perceived dominance and voting likelihood was not significant at an average level of SES (*b* = -0.02, *SE* = 0.04, *t* = -0.50, *p* = .619, 95% CI [-0.10, 0.06]) or high levels of SES (+1 SD, *b* = 0.08, *SE* = 0.06, *t* = 1.42, *p* = .157, 95% CI [-0.03, 0.19]).

**(8) Mini Meta-Analysis across Studies 2a, 2b and 3**

To summarize Studies 2a, 2b and 3, we conducted a small-scale fixed-effects meta-analysis following the recommendations by Goh et al. (2016). This analysis is based on the weighting of a mean effect size by sample size. Before conducting the mini-metanalysis using the R package meta (version 5.5.0, Balduzzi et al., 2019), we converted the *t*-values from the three studies to the unstandardized *bs* into Pearson’s correlation coefficients (see e.g. Kashdan & Steger, 2006). For the analysis, correlations were Fisher’s z-transformed and for presentation of the results, they were converted back to Pearson correlations. For the interaction effect between objective SES and politicians’ perceived competence when controlling for perceived warmth and the interaction between perceived warmth and objective SES, the mini meta-analysis showed a significant effect in the hypothesized direction, *M r* =.14, 95% CI [0.07, 0.20], *Z* = 3.81, *p* < .001. Thus, the effect of perceived competence on voting likelihood was stronger for voters with higher objective SES.

As voters with higher objective SES reported to be significantly more right-leaning in Studies 2a/b and 3 (Studies 2a/b: *r* = .11, *p* = .032, Study 3: *r* = .21, *p* < .001), we ran a parallel analysis across the three separate studies controlling for participants’ political orientation and its interaction with perceived competence as well as with perceived warmth as robustness check. Still the interaction effect of perceived competence and SES remained significant, *M r* =.11, 95% CI [0.04, 0.18], *Z* = 3.12, *p* = .002. Unexpectedly, the interaction effect between perceived competence and political orientation was also significant across the three studies, *M r* =.12, 95% CI [0.05, 0.19], *Z* = 3.46, *p* <.001.

**References**

Adler, N. E., Epel, E. S., Castellazzo, G., & Ickovics, J. R. (2000). Relationship of subjective and objective social status with psychological and physiological functioning: Preliminary data in healthy, White women. *Health Psychology*, *19*(6), 586–592. https://doi.org/10.1037/0278-6133.19.6.586

Balduzzi, S., Rücker, G., & Schwarzer, G. (2019). How to perform a meta-analysis with R: A practical tutorial. *Evidence-Based Mental Health*, *22*(4), 153–160. https://doi.org/10.1136/ebmental-2019-300117

Beierlein, C., Kemper, C. J., Kovaleva, A. & Rammstedt, B. (2012). PEKS. Political Efficacy Kurzskala [Verfahrensdokumentationen mit Items]. In Leibniz-Institut für Psychologie (ZPID) (Hrsg.), Open Test Archive. Trier: ZPID. https://doi.org/10.23668/psycharchives.6561

Beißert, H., Köhler, M., Rempel, M., & Beierlein, C. (2015). Deutschsprachige Kurzskala zur Messung des Konstrukts Need for Cognition NFC-K. *Zusammenstellung sozialwissenschaftlicher Items und Skalen (ZIS).* https://doi.org/10.6102/zis230

Dentler, K., Bluemke, M., & Gabriel, O.W. (2020). German Satisfaction with the Political System Short Scale (SPS). *Zusammenstellung sozialwissenschaftlicher Items und Skalen (ZIS)*. https://doi.org/10.6102/zis278

German Federal Statistical Office (Destatis) (2022). *Bevölkerung und Erwerbstätigkeit. Haushalte und Familien. Ergebnisse des Mikrozensus. Erstergebnisse 2021. Tabelle 1.1.* https://www.destatis.de/DE/Themen/Gesellschaft-Umwelt/Bevoelkerung/Haushalte-Familien/Publikationen/Downloads-Haushalte/haushalte-familien-2010300217004.pdf?__blob=publicationFile

GESIS - Leibniz-Institut für Sozialwissenschaften (2019). Allgemeine Bevölkerungsumfrage der Sozialwissenschaften ALLBUS 2018. *GESIS Datenarchiv, Köln. ZA5270 Datenfile Version 2.0.0, https://doi.org/10.4232/1.13250*

GLES (2022). GLES Querschnitt 2021, Vorwahl. *GESIS, Köln. ZA7700 Datenfile Version 2.0.0.* https://doi.org/10.4232/1.13860

Goh, J. X., Hall, J. A., & Rosenthal, R. (2016). Mini Meta-Analysis of Your Own Studies: Some Arguments on Why and a Primer on How. *Social and Personality Psychology Compass*, *10*(10), 535–549. https://doi.org/10.1111/spc3.12267

Kashdan, T. B., & Steger, M. F. (2006). Expanding the topography of social anxiety. An experience-sampling assessment of positive emotions, positive events, and emotion suppression. *Psychological Science*, *17*(2), 120–128. https://doi.org/10.1111/j.1467-9280.2006.01674.x

Otto, L., & Bacherle, P. (2011). Politisches Interesse Kurzskala (PIKS) – Entwicklung und Validierung. *Politische Psychologie, 1*, 19-35.

1. Among the politicians with the lowest perceived competence three had been used in Study 2a; one of them also in Study 2b. Among the politicians with the highest perceived competence one had been used in Study 2a; two others in Study 2b. [↑](#footnote-ref-1)
